# IL7R remodels immunosuppression tumor microenvironment and promotes macrophage polarization by regulating NF-κB/CXCL1 axis in ovarian cancer

**DOI:** 10.1038/s41419-025-08312-6

**Published:** 2025-12-08

**Authors:** Yeqing Zheng, Cheng Qian, Shuyi Zhang, Meichen Wen, Xun Xu, Xuan Zhou, Yicong Wan, Lin Zhang, Wenjun Cheng

**Affiliations:** Department of Gynecology, the First Affiliated Hospital with Nanjing Medical University, Nanjing, Jiangsu China

**Keywords:** Ovarian cancer, Chemokines, Cancer models, Cancer microenvironment

## Abstract

In the ovarian cancer microenvironment, the polarization of tumor-associated macrophages (TAMs) is closely associated with immunosuppressive phenotypes. Although elevated levels of circulating interleukin-7 (IL-7) have been linked to a poor prognosis, the regulatory mechanisms underlying this association remain incompletely understood. This study demonstrated that increased IL7R expression in ovarian cancer is correlated with the polarization of CD206^+^ macrophages, the remodeling of the immunosuppressive microenvironment, and unfavorable patient prognosis. By employing 3D bioprinted co-culture systems and mouse models, we showed that the knockdown or knockout of IL7R inhibits tumor progression and intraperitoneal dissemination. Mechanistically, IL7R signaling promotes the polarization of macrophages toward an immunosuppressive phenotype through the NF-κB/CXCL1 axis. This is supported by the upregulated expression of Arg1 and IL-10, as well as the downregulated expression of pro-inflammatory markers. These polarized macrophages further enhance tumor cell proliferation and invasion, thereby forming a tumor-immune feedback loop. In conclusion, this study clarifies how IL7R signaling mediates crosstalk between ovarian cancer cells and macrophages to maintain the homeostasis of the immunosuppressive tumor microenvironment (TME).

## Introduction

Ovarian cancer accounts for approximately 2.5% of all malignancies in women worldwide [1]. Due to the insidious nature of its early symptoms, approximately 70% of patients are diagnosed at advanced stages [2, 3]. Current treatment strategies, including surgery, chemotherapy, and targeted therapy, are customized based on tumor stage, histopathological type, molecular characteristics, and patient conditions [4–6]. Despite the availability of multiple treatment methods, the prognosis of ovarian cancer remains poor; the 5-year survival rate of patients with advanced-stage ovarian cancer is approximately 40% [7]. This unfavorable prognosis is primarily attributed to factors such as tumor cell drug resistance and the immunosuppressive nature of the tumor microenvironment (TME) [8, 9].

The TME is a complex ecosystem composed of blood vessels, immune cells, and fibroblasts [10]. Through direct cell-cell contact and paracrine signaling, tumor cells remodel the surrounding stroma into a TME that supports tumor progression [10]. Concurrently, the accumulation of regulatory T cells (Tregs) and tissue-resident macrophages within the TME establishes a suppressive microenvironment, enabling tumor immune evasion [10, 11]. Previous studies have reported that cytokines secreted by cancer-associated fibroblasts (CAFs) enhance the chemoresistance of tumor cells. Additionally, TMEs enriched with CAFs and M2-type tumor-associated macrophages impede the infiltration of CD8^+^ T cells, thereby further facilitating tumor immune evasion [12, 13]. Notably, ovarian cancer was among the first human cancers in which a positive association was identified between higher intraepithelial tumor-infiltrating lymphocyte (TIL) density and longer patient survival. This finding underscores the critical role of the TME in regulating the biological behaviors of ovarian cancer cells [14, 15].

Interleukin-7 (IL-7) is primarily secreted by the intestinal epithelium, lymph nodes, and thymic stroma [16]. The regulatory role of IL-7 in immune cells—mediated by its binding to the receptor IL7R—has been extensively studied, and emerging evidence indicates that IL-7/IL7R signaling modulates tumor-immune interactions [17, 18]. Kast et al. analyzed serum samples from 187 ovarian cancer patients and observed that serum IL-7 levels were significantly higher in patients with advanced-stage ovarian cancer (FIGO III/IV) than in those with early-stage disease (I/II) [19, 20]. In contrast, Xie et al. detected no IL-7 protein expression in tumor epithelial cells [21]. This discrepancy suggests that serum IL-7 in ovarian cancer patients may originate from other cell types within the tumor microenvironment (TME; e.g., immune cells or stromal cells) or be indirectly produced via systemic inflammatory responses. Currently, the specific mechanisms underlying the functions of IL-7 and its receptor (IL7R) in the ovarian cancer TME remain unclear. Further investigations are required to clarify their sources, regulatory networks, and associations with tumor immune evasion.

Although conventional preclinical 2D cell culture models are widely used in cancer research, they often fail to replicate the inherent complexity of tumors [22]. Ovarian tumors exhibit a characteristic spatial architecture with tumor cells embedded within the surrounding TME [23]. Reconstructing these cellular interactions in artificial in vitro environments remains challenging: 2D cultures cannot achieve the appropriate spatial arrangement of specific cell populations [24], while 3D spheroid models—typically composed of single cell type or simple co-cultures (e.g., tumor cells + fibroblasts)–struggle to mimic multicellular interactions. Additionally, conventional 3D spheroid models are unable to sustain the long-term functional infiltration and functional activity of immune cells [25, 26]. Therefore, 3D bioprinting has emerged as a promising technology, enabling the precise, automated spatial placement of diverse cellular components to closely simulate the heterogeneity of the ovarian cancer TME [27–29].

In this study, a physiologically relevant 3D bioprinted platform and animal models were used to demonstrate that ovarian cancer cells expressing IL7R induce remodeling of the tumor immune microenvironment and promote the polarization of macrophages toward an anti-inflammatory phenotype.

## Results

### Ovarian cancer cells express IL7R and drive tumor progression

**Fig. 1:**
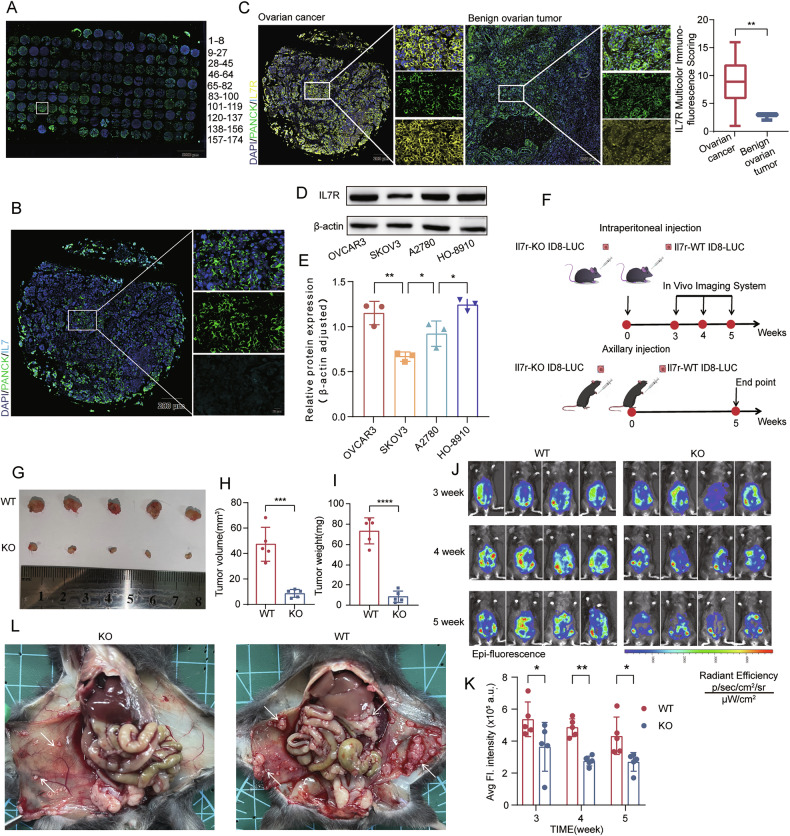
IL7R expression and functional validation in ovarian cancer models. **A** Representative multiplex immunofluorescence images of a tissue microarray (TMA) with 174 ovarian cancer samples, showing co-expression of IL-7 (light blue) and the epithelial marker PANCK (green). Nuclei were counterstained with DAPI (dark blue). Overview image acquired at ×0.2 magnification (scale bar: 5000 µm); **B** enlarged views of boxed regions in (**A**), highlighting tumor areas at ×2 (left; scale bar: 200 µm) and ×20 (right; scale bar: 20 µm) magnifications; **C** comparative multiplex immunofluorescence staining of IL7R (yellow) and PANCK (green) in ovarian cancer (left) (N = 174) and benign ovarian tumor tissues (right) (N = 3). Nuclei were stained with DAPI (dark blue). Images captured at ×2 and ×20 magnifications (scale bars: 200 µm and 20 µm, respectively); **D**, **E** western blot analysis of IL7R expression in four human ovarian cancer cell lines (N = 3); **F** schematic of the experimental design. *Il7r*-WT or *Il7r*-KO ID-8 cells were injected subcutaneously (left flank) or intraperitoneal (metastasis model) into mice. In the metastatic cohort, bioluminescence imaging was performed weekly starting at Week 3; subcutaneous tumors were excised at Week 5; subcutaneous tumor characterization: **G** representative images, **H** tumor volume dynamics, and **I** tumor weight at harvest (N = 5 mouse/group); metastatic burden assessment: **J** representative bioluminescent images and **K** quantitative analysis of fluorescence intensity (N = 5 mouse/group); **L** macroscopic metastatic nodules (white arrows) in the peritoneal cavity, mesentery, and intestinal surfaces in the metastasis model. Statistical significance: ns (not significant), *P < 0.05, **P < 0.01, ***P < 0.001, ****P < 0.0001. Data are presented as mean ± SEM.

Previous studies have established that serum is the main source of human IL-7. To evaluate the expression of IL-7 and IL7R in ovarian cancer tissues, immunofluorescence staining was performed on human tissue microarrays. IL-7 expression was undetectable or low in most ovarian cancer epithelial cells (Fig. 1A, B), which is consistent with the results of a previous small-cohort study by Xie et al. [21]. This suggests that IL-7 does not originate from tumor cells or their microenvironment. Subsequently, this study measured the concentrations of IL-7 in the plasma and ascites of ovarian cancer patients, and compared them with the concentrations of IL-7 in the plasma and peritoneal lavage fluid of patients with benign ovarian tumors. The results showed that the levels of IL-7 in the plasma and ascites of ovarian cancer patients were elevated (Fig. S1A；clinicopathological details are provided in Supplementary Table S1). Combined with the low expression of IL-7 in the tissue microarrays, these results further confirm the hypothesis of the study—that IL-7 may primarily originate from the systemic circulation.

IL-7 exerts its biological effects primarily through its receptor IL-7R [30]. We further investigated the expression of IL7R in ovarian cancer cells and found that IL7R levels were significantly upregulated in malignant tissues compared with those in benign ovarian tumors, with staining mainly localized in tumor cells (Fig. 1C; clinicopathological details are provided in Supplementary Table S1). Additionally, IL7R was stably expressed across four human ovarian cancer cell lines (Fig. 1D, E), indicating that these cells may be responsive to circulating IL-7.

To assess the role of IL7R in ovarian cancer progression, CRISPR-Cas9 technology was used to generate *Il7r*-knockout (*Il7r*-KO) ID-8 mouse ovarian cancer cell lines. The knockout efficiency was verified by western blotting and Sanger sequencing (Supplementary Fig. S1B, C). Subcutaneous tumors models and intraperitoneal metastasis models were then established using these cells (Fig. 1F). In the subcutaneous xenograft model, *Il7r*-KO cells exhibited significantly suppressed tumor growth (Fig. 1G–I and Supplementary Fig. S1D). In the intraperitoneal metastasis model, continuous bioluminescence imaging showed that mice inoculated with *Il7r*-KO cells had a reduced tumor burden compared with mice inoculated with *Il7r*-wide-type (*Il7r*-WT) cells (Fig. 1J, K), along with fewer peritoneal metastatic nodules (Fig. 1L and Supplementary Fig. S1E)—especially in common metastatic sites such as the peritoneum, intestinal tract, and diaphragm. The formation of hemorrhagic ascites was also reduced (Supplementary Fig. S1E). These results demonstrated that IL7R signaling is critical for the tumorigenicity and intraperitoneal dissemination of ovarian cancer cells.

### IL7R regulates tumor-stroma interactions to drive ovarian cancer progression in 2D and 3D models

In 2D culture systems, *Il7r*-KO cells displayed reduced clonogenic and migratory capacities compared with the WT cells (Supplementary Fig. S2A–C). Exogenous supplementation with recombinant IL-7 significantly enhanced these functions in WT cells (Supplementary Fig. S2D–F), while *Il7r*-KO cells showed no response to IL-7 stimulation (Supplementary Fig. S2D–F); this confirms an IL7R-dependent regulatory mechanism.

**Fig. 2:**
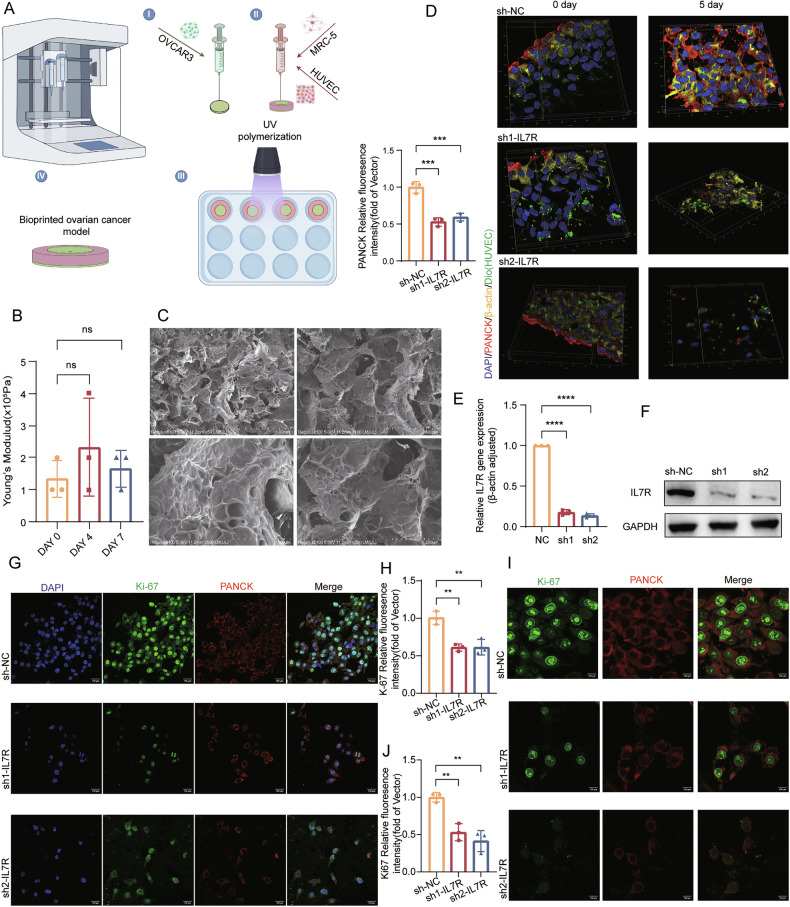
3D bioprinted tumor-stroma co-culture model and IL7R functional analysis. **A** Schematic representation of 3D bioprinting strategy. A dual-nozzle system generated concentric constructs: inner ring: GelMA-embedded OVCAR3 cells (tumor compartment); outer ring: GelMA-embedded stromal components (MRC-5 + HUVECs). The constructs were cross-linked via photopolymerization and cultured for functional assays. The images sourced from Figdraw: **B** time-dependent changes in Young’s modulus of the hydrogels measured on days 0, 4, and 7 (N = 3); **C** scanning electron microscopy (SEM) images of GelMA hydrogels demonstrating a uniform porous architecture (N = 1). Scale bars (from top left, clockwise): 1000, 500, 200, and 100 µm; **D** 3D-reconstructed multiplex immunofluorescence of tumor-stroma co-culture (N = 3): day 0: distinct boundary between PANCK^+^ tumor cells (red) and stromal components. β-actin (yellow), HUVECs labeled with DiO (green), and MRC-5 cells identified as DAPI^+^/β-actin^+^/PANCK^−^/DiO^−^. Day 5: Tumor cell migration was assessed by PANCK^+^ signal infiltration into stromal regions. The nuclei were stained with DAPI (blue). High-magnification (×60) view; validation of IL7R knockdown (KD) in OVCAR3 cells (N = 3): **E** qPCR analysis of IL7R mRNA expression; **F** Western blot analysis of IL7R protein levels; multiplex immunofluorescence imaging on day 5 of co-culture (N = 3): **G** low-magnification (×40) overview showing PANCK^+^ tumor cells (red), proliferating cells (Ki-67, green), and nuclei (DAPI, blue); **H** quantification of ×40 fluorescence intensity; **I** high-magnification (×100) view of the invasive front (N = 3); and **J** quantification of ×100 fluorescence intensity. Scale bar: 100 µm. Statistical significance: ns (not significant), *P < 0.05, **P < 0.01, ***P < 0.001, ****P < 0.0001. Data are presented as mean ± SEM.

To better recapitulate tumor-stroma interactions, a 3D bioprinted model was developed using a precision extrusion bioprinting system. The core region contained ovarian cancer cells encapsulated in 7% GelMA hydrogel, whereas the peripheral stroma was composed of vascular endothelial cells (HUVECs) and fibroblasts (MRC-5) in a 1:1 ratio (Fig. 2A)—a design that mimics vascular and mesenchymal signaling pathways. Rheological analysis revealed stable mechanical properties over 7 days, with a constant Young’s modulus (Fig. 2B). Transmission electron microscopy (TEM) images showed a uniformly distributed and interconnected microporous network within the 10 × 10 × 10 mm cubic structure (Fig. 2C and Supplementary Fig. S2G; Video 1), which facilitates the diffusion of nutrients and signaling molecules. Cell viability remained above 90% (Supplementary Fig. S2H), verifying that the model is capable of supporting multicellular co-cultures.

Immunofluorescence staining showed a clear spatial boundary between PANCK-positive tumor cells and DiO-prelabeled HUVECs (Fig. 2D). To investigate the function of IL7R, stable IL7R-knockdown ovarian cancer cell lines were established using lentiviral shRNA; qPCR and western blotting confirmed the downregulated expression of IL7R at both the mRNA and protein levels (Fig. 2E, F). These IL7R-knockdown cells were co-bioprinted with stromal components to construct a 3D tumor-stroma model (Supplementary Fig. S2H, Video 2). After 5 days of co-culture, IL7R knockdown significantly inhibited cell proliferation, as evidenced by decreased cell density and reduced Ki-67 positivity (Fig. 2G–J). Dynamic tracking at the tumor-stroma interface demonstrated that IL7R knockdown shortened the migration distance of tumor cells from the printing boundary into the stroma (Fig. 2D).

### Tumor cells expressing IL7R reshape the immune microenvironment and correlate with poor prognosis in ovarian cancer

**Fig. 3:**
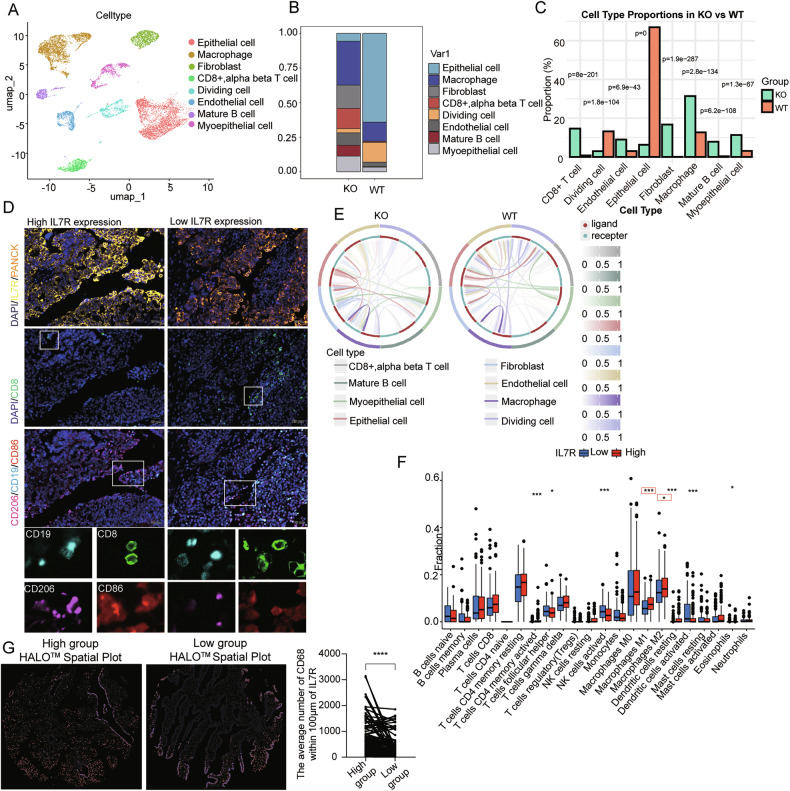
Single-cell and spatial analysis of IL7R-associated tumor-immune interactions in ovarian cancer. **A** UMAP projection of integrated single-cell transcriptomic data from mouse ovarian cancer tissues (N = 1), identifying distinct cellular clusters; **B** distribution of cellular clusters across individual mouse ovarian cancer samples; **C** stacked bar plot showing the percentage of major cell types in *Il7r*-KO and *Il7r*-WT samples, P values were calculated via Fisher’s exact test; **D** consecutive sections of human ovarian cancer tissue microarray (TMA) analyzed by multiplex immunofluorescence: Section 1: IL7R (yellow) and epithelial marker PANCK (orange); Section 2: T cell marker CD8 (green); Section 3: Macrophage marker CD68 (red), immunosuppressive-polarized macrophage marker CD206 (pink), and B cell marker CD19 (light blue). Nuclei were counterstained with DAPI (blue). Left panel: Tumor tissue from an IL7R-high patient with sparse immune cell infiltration in the stroma. Right panel: Tumor tissue from an IL7R-low patient with abundant immune cell infiltration. Images acquired at ×20 magnification (scale bar: 20 µm); **E** circos plot depicting cell-cell interaction networks: Left: IL7R-KO group; Right: IL7R-WT group. Gray ribbons connect interacting cell types, with ribbon color intensity reflecting interaction strength (analyzed by the CellCall algorithm); **F** immune cell infiltration scores estimated by CIBERSORT in TCGA-OV samples, stratified based on IL7R expression (high vs. low); **G** HALO spatial analysis of CD68 co-expression-positive cells within a 100-µm radius of tumor cells in ovarian cancer TMA sections: Comparison between IL7R-high and IL7R-low groups.

To characterize the role of IL7R in the ovarian cancer TME, single-cell RNA sequencing (scRNA-seq) was performed on tumor tissues derived from mouse models inoculated with *Il7r*-WT cells and *Il7r*-KO cells. After quality control, the cells were clustered into eight major cell subpopulations (Fig. 3A and Supplementary Fig. S3A) and annotated using canonical molecular markers (Fig. S3B). Tumors deficient in Il7r exhibited significant alterations in immune cell infiltration: the proportions of mature B cells and CD8^+^ T cells were increased, while macrophages infiltration was tripled (Fig. 3B). In contrast, WT tumors were predominantly composed of malignant epithelial cells, reflecting the “immune desert” phenotype that is typical of ovarian cancer (Fig. 3B). We further quantified the differences in cell type proportions between tumor tissues of mice inoculated with *Il7r*-KO cells and those with *Il7r*-WT cells. The results showed that there were statistically significant differences in the proportions of multiple cell types (Fig. 3C). These results indicated that Il7r deficiency remodels the ovarian cancer TME to promote immune cell infiltration. Multiplex immunofluorescence staining of human ovarian cancer tissues revealed that tumors with high Il7r expression showed restricted immune cell infiltration, which was mainly localized at the tumor periphery, with minimal immune cells present in the tumor core (Fig. 3D). The abundances of key immune subsets, including CD8^+^ T cells, B cells, and macrophages, were significantly lower in tumors with high Il7r expression.

Tumor-infiltrating immune cells (TIICs) are core components of the TME that dynamically regulate tumor progression. To investigate the mechanisms underlying the interaction between tumor cells and immune cells, we analyzed cell communication networks using CellCall. We found that in the *Il7r*-regulated TME, tumor cells exhibited stronger interactions with macrophages than with CD8^+^ T or B cells (Fig. 3E). Analysis of the TCGA-OV dataset showed that *IL7R* expression was positively correlated with the pan-macrophage markers CD68 and CD206^+^ macrophages and the CD8^+^ T marker CD8, but no significant correlation was observed with the B cell marker CD19 (Supplementary Fig. S3C). CIBERSORT deconvolution confirmed that the infiltration of both M1 and M2 macrophages was increased in *IL7R*-high tumors, while there was no significant association with the infiltration of CD8^+^ T or B cells (Fig. 3F). In addition, after performing differential analysis on B cells in the single-cell data, we found that there were only 3 differentially expressed genes, namely Igfbp2, Rpl24, and Dusp1, none of which are associated with the major functions of B cells (Supplementary Fig. S3D) [31–33]. For CD8 + T cells, differential analysis identified only 12 differentially expressed genes, including Btg1, Junb, and Fos, which are related to cellular stress; no gene differences associated with the functions of CD8+ T cells were observed (Supplementary Fig. S3E) [34]. Multicolor immunofluorescence staining was independently scored by two clinical pathologists; the results indicated that *Il7r*-high tumors showed increased infiltration of CD206^+^ macrophage, whereas no such increase was observed for CD8^+^ T cells, B cells, and CD86^+^ macrophages did not (Supplementary Fig. S3F; scoring criteria are provided in Supplementary Table S2). HALO quantitative analysis showed that compared with *IL7R*-low tumors, *IL7R*-high tumors exhibited significantly more frequent colocalization (defined as positive co-expression) between tumor cells and macrophages (Fig. 3G), which supports the hypothesis that tumor cells expressing IL7R promote macrophage recruitment. Thus, we speculate that ovarian cancer cells expressing IL7R have a close association with tumor-associated macrophages in the immune microenvironment.

We subsequently analyzed the correlation between IL7R expression and progression-free survival (PFS) in early-stage and late-stage ovarian cancer patients who had undergone optimal debulking surgery. The results showed that there was no significant difference in PFS between the IL7R-high expression group and the IL7R-low expression group among early-stage patients; however, in late-stage patients, the PFS of the IL7R-high expression group was significantly shorter than that of the IL7R-low expression group. This suggests that high IL7R expression is a potent predictor of PFS in late-stage patients who have undergone optimal debulking surgery, and clinical management should be stratified according to tumor stage. Meanwhile, we analyzed the correlation between IL7R expression and PFS in patients with low-grade and high-grade ovarian cancer who had undergone optimal debulking surgery. The results showed that regardless of whether the tumor was low-grade or high-grade, the progression-free survival probability of the IL7R-high expression group was significantly lower than that of the IL7R-low expression group; moreover, the statistical significance of this difference was more pronounced in high-grade patients. This indicates that high IL7R expression may be an adverse prognostic indicator for the shortened PFS of such patients (Supplementary Fig. S3G). Collectively, these findings demonstrate that *IL7R* signaling in ovarian cancer cells drives the remodeling of the immunosuppressive microenvironment and promotes the polarization of macrophages toward an immunosuppressive state (characterized by the CD206^+^ phenotype).

### Tumor cells expressing IL7R promote immunosuppressive state of tumor-associated macrophages

**Fig. 4:**
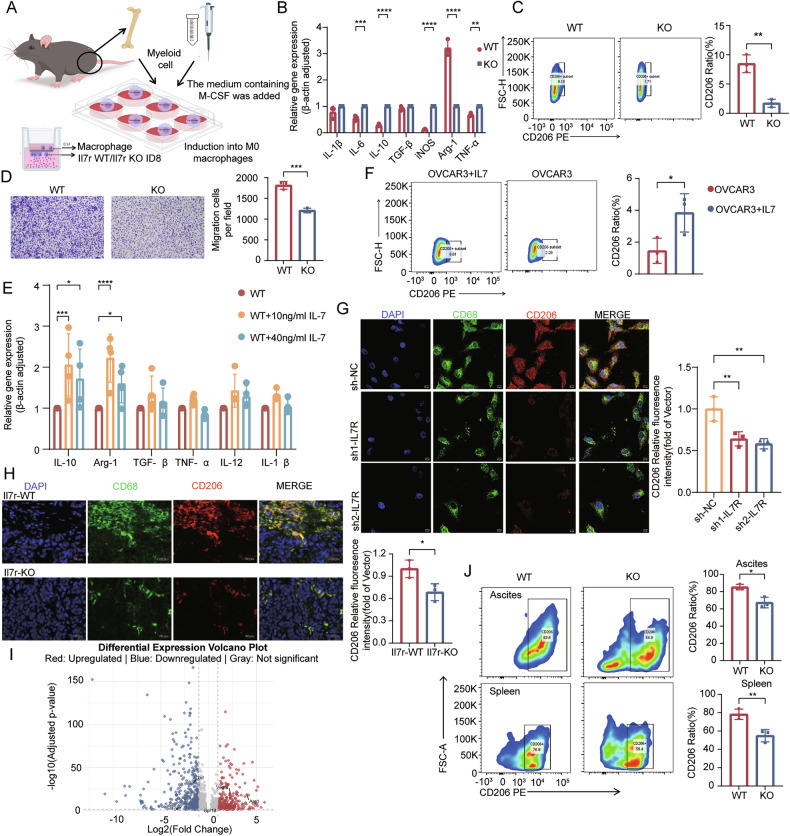
IL7R modulates macrophage polarization and migration in ovarian cancer. **A** Schematic of the in vitro co-culture system between bone marrow-derived macrophages (BMDMs) and tumor cells. Images sourced from Figdraw; **B** qPCR analysis of macrophage polarization markers in BMDMs co-cultured with *Il7r*-KO murine ovarian cancer cells versus WT controls (N = 3); **C** flow cytometry quantification of macrophage polarization in BMDMs co-cultured with *Il7r*-KO or WT tumor cells (N = 3); **D** Transwell migration assay assessing chemotaxis of BMDMs toward *Il7r*-KO or WT tumor cells (N = 3); **E** qPCR analysis of macrophage polarization markers after adding recombinant IL7 protein to WT murine ovarian cancer cells (N = 3); **F** flow cytometry analysis of macrophage polarization in human ovarian cancer cells treated with exogenous recombinant IL7 (N = 3); **G** 3D bioprinted co-culture of M0-polarized THP-1 cells with OVCAR3 cells for 5 days, followed by multiplex immunofluorescence to assess the impact of IL7R-knockdown OVCAR3 cells on macrophage polarization (CD68: green; CD206: red; nuclei/DAPI: blue) (N = 3). Scale bar: 20 µm; **H** multicolor immunofluorescence was performed to verify the expression of tumor-associated macrophage markers in ovarian cancer tissues from mice injected with *Il7r*-WT and *Il7r*-KO cells (CD68: green; CD206: red; nuclei/DAPI: blue) (N = 3). Scale bar: 20 µm; **I** volcano plot of differentially expressed genes in TAMs from *Il7r*-KO versus WT murine ovarian tumors (single-cell RNA sequencing); **J** flow cytometry analysis of CD206^+^ TAM proportions in ascites and spleens of mice bearing *Il7r*-WT or *Il7r*-KO ID8 tumors (N = 3). Statistical significance: ns (not significant), *P < 0.05, **P < 0.01, ***P < 0.001, ****P < 0.0001. Data represent mean ± SEM.

To systematically investigate the impact of *IL7R*-expressing tumor cells on TAM polarization, an in vitro co-culture system was established using bone marrow-derived macrophages (BMDMs), taking into account the potential confounding effects of systemic factors in in vivo models (Fig. 4A). Based on the significant correlation between IL7R and CD206+ macrophages, this section of experiments focused on verifying the macrophage polarization phenotype. The selected markers were all well-recognized signature molecules for pro-inflammatory or anti-inflammatory macrophages. CD206 is a specific surface marker of anti-inflammatory macrophages; Arg1 and IL-10 are typical immunosuppressive factors secreted by anti-inflammatory macrophages, while iNOS, IL-6, and TNF-α are hallmark pro-inflammatory molecules. By comparing the expression changes of these markers, the polarization direction of macrophages can be clearly reflected [35]. BMDMs co-cultured with *Il7r*-WT cells showed upregulated *Arg1* expression and decreased levels of proinflammatory cytokines (*Il6, Nos2, Tnf*) (Fig. 4B), along with a significant increase in the proportion of CD206^+^ macrophages (Fig. 4C). Transwell migration assays demonstrated that WT tumor cells significantly enhanced BMDM chemotaxis (Fig. 4D).

Recombinant IL-7 was exogenously added to the co-culture system to dissect the IL-7/IL7R signaling axis. IL-7 alone had no effect on BMDM polarization (Supplementary Fig. S4A, B), but it further upregulated the expression of *Il10* and *Arg1* in macrophages co-cultured with WT tumor cells (Fig. 4E); no such response was observed in macrophages co-cultured with *Il7r*-KO tumor cells (Supplementary Fig. S4C). This indicated that exogenous IL-7 could enhance the ability of WT tumor cells to induce the immunosuppressive state of macrophages, and this phenomenon was validated in the human OVCAR3/THP-1 co-culture model (Fig. 4F and Supplementary Fig. S4D). To better simulate the in vivo TME, 3D bioprinting technology was used to construct a composite model containing tumor cells, stromal cells, and THP-1 cells. Immunofluorescence staining revealed that the regions with IL7R-expressing tumor were specifically enriched with CD68^+^CD206^+^ macrophages (Fig. 4G). Subsequently, consistent results were also observed in ovarian cancer tissues of mice inoculated with *Il7r*-WT or *Il7r*-KO cells: specifically, the expression of CD206 in TAMs was significantly enhanced in the TME of ovarian cancer with IL7R-expressing tumor cells (Fig. 4H). This result was consistent with the findings from the 3D bioprinted model, confirming that the 3D model can effectively simulate the characteristics of the in vivo immune microenvironment.

Additionally, transcriptomic analysis of TAMs from scRNA-seq data showed that TAMs in the TME of mice inoculated with *Il7r*-WT cells highly expressed immunosuppressive signature genes (*Arg1* and *Vegf*) and had downregulated pro-inflammatory markers (*Gpr18*, *Fpr2*, *Tnf*) (Fig. 4I; Supplementary Table S3), which reflects an immunosuppressive tumor phenotype [36]. KEGG pathway enrichment analysis identified significant activation of the HIF-1 signaling pathway in TAMs from the TME of mice inoculated with *Il7r*-WT cells; this pathway is crucial for the immunosuppressive function of macrophages and tumor progression (Supplementary Fig. S4E) [37]. To confirm whether these gene expression differences translate into actual cellular phenotypic changes, flow cytometry was used to analyze the distribution of macrophage subsets in tumor-bearing mouse models. Flow cytometry results confirmed that the proportion of CD206^+^ macrophages was higher in mice inoculated with *Il7r*-WT cells than in those inoculated with *Il7r*-KO cells (Fig. 4J; the gating strategy is shown in Supplementary Fig. S4F).

In human ovarian cancer tissues, regions with high IL7R expression were significantly enriched in CD206^+^ TAMs (Fig. 3F). In summary, *IL7R*-expressing ovarian cancer cells promote the immunosuppressive state of TAMs, thereby facilitating the establishment of an immunosuppressive microenvironment.

### IL7R-expressing tumor cells promote macrophage polarization via NF-κB-mediated CXCL1 secretion

**Fig. 5:**
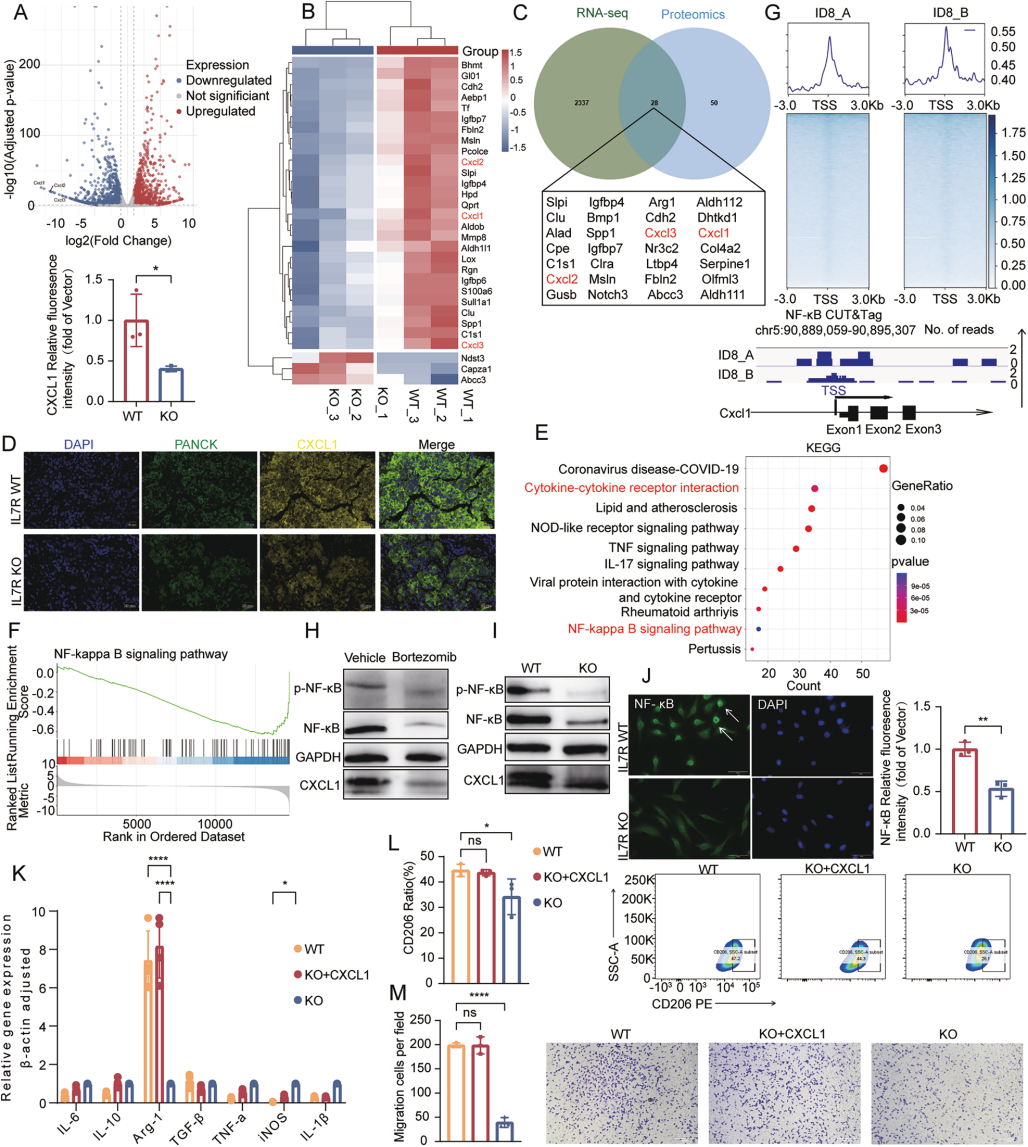
Multi-omics analysis reveals *Il7r*-KO-driven transcriptional and proteomic reprogramming via NF-κB/CXCL1 axis. **A** Volcano plot of differentially expressed genes (DEGs) in IL7R-KO versus IL7R-WT cells (N = 3 vs. 3). Downregulated and upregulated genes are highlighted in blue and red, respectively; **B** heatmap of 30 significantly altered proteins identified by deep proteomics analysis, showing hierarchical clustering of expression patterns (N = 3 vs. 3); **C** Venn diagram depicting overlap between RNA-seq-derived DEGs and proteomics-identified differentially expressed proteins, highlighting concordant molecular changes at the transcriptomic and proteomic levels; **D** multiplex immunofluorescence of tumor tissues stained for PANCK (green, epithelial cells), CXCL1 (yellow), and DAPI (blue, nuclei) (N = 3). Representative ×20 images (scale bar: 20 µm) show reduced CXCL1 expression in IL7R-KO tumor sections compared to WT controls; KEGG pathway enrichment (**E**) and GSEA (**F**) analyses of DEGs in IL7R-KO cells, identifying NF-κB signaling and cytokine-related pathways as top functional categories; **G** CUT&Tag profiling of NF-κB binding sites in ID8-WT cells (N = 2). Density heatmap illustrates NF-κB occupancy within ±3.0 kb of transcription start sites (TSS). Representative genomic tracks (bottom) confirm NF-κB binding at the CXCL1 locus (blue boxes denote peak regions); Western blot analysis of total NF-κB, phospho-NF-κB (p-p65), and CXCL1 protein levels in: **H** IL7R-WT (N = 3) cells treated with NF-κB inhibitor (15 µM, 24 h) versus vehicle control (N = 3); **I** IL7R-KO versus IL7R-WT cells. GAPDH served as a loading control; **J** IF staining for p65 nuclear translocation (arrows indicate cells with nuclear p65 localization) (N = 3). Images acquired at ×20 magnification (scale bar: 100 µm); **K** flow cytometry analysis of immunosuppressive macrophage polarization in bone marrow-derived macrophages (BMDMs) co-cultured with murine *Il7r*-KO ovarian cancer cells treated with or without CXCL1 and *Il7r*-WT tumor cells (N = 3); **L** qPCR validation of macrophage polarization marker genes in BMDMs co-cultured with CXCL1-treated *Il7r*-KO tumor cells, *Il7r*-KO tumor cells and *Il7r*-WT tumor cells (N = 3); **M** Transwell migration assay to assess chemotaxis of BMDMs toward conditioned media from CXCL1-treated or untreated murine *Il7r*-KO ovarian cancer cells and *Il7r*-WT tumor cells. Migrated cells were quantified after 24 h of incubation (N = 3). Scale bar: 100 µm. Statistical significance: ns (not significant), *P < 0.05, **P < 0.01, ***P < 0.001, ****P < 0.0001. Data are presented as mean ± SEM.

To dissect the molecular mechanisms underlying IL7R-regulated TAM polarization, RNA-sequencing (RNA-seq) was performed on *Il7r*-WT and *Il7r*-KO tumor cells, which led to the identification of 2674 differentially expressed genes (Fig. 5A**;** Supplementary Table S4). Considering the role of secreted factors in cell-cell communication, quantitative proteomic analysis was conducted on conditioned media, resulting in the detection of 66 differentially expressed proteins (Fig. 5B; Supplementary Table S5). Integration of transcriptomic and proteomic datasets identified 28 potential regulatory genes, among which the expression levels of *Cxcl1*, *Cxcl2* and *Cxcl3* were significantly downregulated in *Il7r*-KO cells (Fig. 5C). This finding was consistent with the analysis results from the TISIDB database, which showed positive correlations between *IL7R* and *CXCL1/CXCL2/CXCL3* expression in human ovarian cancer tissues (Supplementary Fig. S5A).

To verify whether these candidate molecules serve as key downstream genes regulated by *IL7R* signaling, qPCR was performed. The results confirmed that the mRNA expression levels of *Cxcl1* and *Cxcl2* were decreased in *Il7r*-KO tumor cells, while the expression of *Cxcl3* remained unchanged (Supplementary Fig. S5B). Thus, *Cxcl1* and *Cxcl2* were prioritized as candidate effectors. In mouse models, the protein level of *Cxcl1* was significantly lower in mice injected with *Il7r*-KO cells (Fig. 5D), whereas no significant difference was observed in *Cxcl2* levels (Supplementary Fig. S5C).

To clarify the mechanism by which IL7R regulates CXCL1 expression, pathway enrichment analysis was performed on the transcriptome sequencing data. Interestingly, although the classical JAK/STAT and PI3K/AKT pathways (downstream of *IL7R* signaling) were not significantly enriched [30], the NF-κB pathway was significantly enriched in *Il7r*-WT cells (Fig. 5E, F). Three transcription factor databases (CISBP, HOCOMOCO, and hTFtarget) were integrated to predict the transcription factors that regulate *Cxcl1*, leading to the identification of 55 potential common transcription factors (Supplementary Fig. S5D). Among these transcription factors, RelA/p65—a classical transcriptional regulator of the NF-κB pathway—was significantly enriched in the promoter regions of differentially expressed genes. This result suggests that RelA/p65 may play a role in mediating *IL7R*-dependent transcriptional regulation of *Cxcl1*. To test this hypothesis, CUT&Tag sequencing was performed to analyze whether NF-κB (RelA/p65) binds to the promoter region of the *Cxcl1* gene. Within 1 kb upstream region of the *Cxcl1* transcription start site (TSS) (chr4:73868995-73869451), a 457 bp enrichment peak was identified (Fig. 5G), indicating that NF-κB signaling can directly regulate *Cxcl1* expression. For further validation, the NF-κB inhibitor bortezomib was used, and it significantly reduced CXCL1 expression (Fig. 5H). Additionally, *Il7r*-KO cells exhibited significantly decreased levels of total NF-κB protein, phosphorylated p65, and CXCL1 (Fig. 5I). Immunofluorescence analysis confirmed that *Il7r*-KO significantly reduced the nuclear translocation of p65 (Fig. 5J).

To further verify that CXCL1 is the key factor mediating the phenotypic changes of macrophages induced by tumor cells, recombinant CXCL1 protein was added to the co-culture system of Il7r-KO cells and macrophages. The results showed that after the exogenous addition of CXCL1, the migration number of macrophages and the expression levels of CD206 and Arg1 were restored (Fig. 5K–M). Meanwhile, a similar phenomenon was observed after the co-culture of IL7R-knockdown OVCAR3 cells and THP-1-induced macrophages (Fig. S5E). Taken together, these results indicate that *IL7R* drives *CXCL1* expression through NF-κB signaling, thereby promoting immunosuppressive macrophage polarization.

### IL7R promotes TAM polarization toward immunosuppressive phenotype and ovarian cancer progression through the NF-κB-CXCL1-CXCR2 axis

**Fig. 6:**
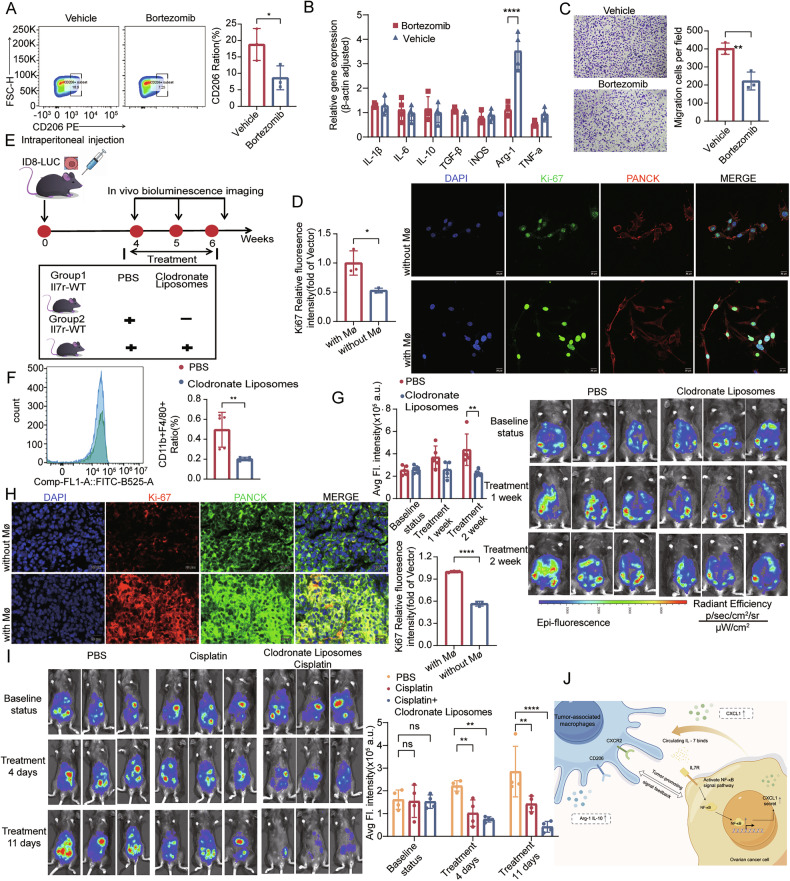
NF-κB-dependent regulation of macrophage polarization and migration by ovarian cancer cells, and therapeutic effects of macrophage depletion. **A** Flow cytometry analysis of immunosuppressive macrophage polarization in bone marrow-derived macrophages (BMDMs) co-cultured with murine ovarian cancer cells treated with or without an NF-κB inhibitor (15 µM) (N = 3); **B** qPCR validation of macrophage polarization marker genes in BMDMs co-cultured with NF-κB inhibitor-treated tumor cells (N = 3); **C** Transwell migration assay to assess chemotaxis of BMDMs toward conditioned media from NF-κB inhibitor-treated or untreated murine ovarian cancer cells. Migrated cells were quantified after 24 h of incubation (N = 3). Scale bar: 100 µm; **D** 3D bioprinted co-culture of immunosuppressive-polarized THP-1 cells with OVCAR3 cells for 5 days, followed by multiplex immunofluorescence to evaluate immunosuppressive macrophage effects on tumor cell proliferation: Ki-67 (green, proliferative marker); PANCK (red, epithelial marker); nuclei stained with DAPI (blue) (N = 3). Scale bar: 20 µm; **E** schematic of clodronate liposome treatment in an ID8-LUC murine ovarian cancer model. Mice were intraperitoneally injected with ID8-LUC cells, and baseline tumor burden was assessed by bioluminescence imaging (BLI) at Week 4. Treatment commenced at Week 4 with intraperitoneal injections of PBS (control) or clodronate liposomes (10 mg/kg, twice weekly). BLI was repeated at Weeks 5 and 6 to monitor metastatic progression. Images sourced from Figdraw; **F** flow cytometry analysis of macrophage depletion efficiency in spleens of clodronate liposome-treated mice, quantified by CD11b^+^ F4/80^+^ cell frequency (gated on live cells) (N = 5 mouse/group); **G** metastatic burden assessment: Representative BLI images of mice from PBS- and clodronate liposome-treated groups at Week 6, depicting abdominal bioluminescence intensity (N = 5 mouse/group); **H** multicolor immunofluorescence was conducted to verify the expression of tumor cell-related markers in mouse ovarian cancer tissues under conditions of macrophage depletion or non-depletion (PANCK: green; Ki-67: red; nuclei/DAPI: blue) (N = 3). Scale bar: 20 µm; **I** metastatic burden assessment: Representative BLI images of mice from PBS, Cisplatin, and clodronate liposomes, combining with Cisplatin groups, depicting abdominal bioluminescence intensity (N = 4 mice/group); **J** signal pathway diagram; image sourced from Figdraw. Statistical significance: ns (not significant), *P < 0.05, **P < 0.01, ***P < 0.001, ****P < 0.0001. Data are presented as mean ± SEM.

Previous studies have confirmed that CXCL1 primarily exerts its signaling function through the C-X-C Motif Chemokine Receptor 2 (CXCR2) to regulate various inflammatory cell functions [38, 39]. To investigate the role of the IL7R-NF-κB-CXCL1-CXCR2 axis in macrophage polarization, an in vitro coculture system was utilized. Pretreatment with bortezomib, an NF-κB inhibitor, significantly reduced the expression of CD206 (an immunosuppressive marker) in macrophages (Fig. 6A), decreased the mRNA expression of *Arg-1* (Fig. 6B), and inhibited macrophage migration (Fig. 6C). To specifically block the CXCL1-CXCR2 interaction, Navarixin (a CXCR2 antagonist) was added to the co-culture system. This treatment resulted in a significant downregulation of CD206 expression in TAMs, a reduction in the mRNA expression of anti-inflammatory factors (*Arg-1* and *Il-6*), and impairment of macrophage migration (Supplementary Fig. S6A–C).

To explore the feedback regulation of macrophage polarization on the malignant behaviors of tumor cells, 3D bioprinting technology was employed to construct a co-culture system of tumor cells and macrophages. Immunosuppressively polarized macrophages significantly promoted the proliferation of OVCAR3 tumor cells, which was evidenced by the increased epithelial cell density and enhanced Ki67 fluorescence signal intensity (Fig. 6D). This finding confirmed that immunosuppressive TAMs can facilitate the progression of ovarian cancer. Additionally, a syngeneic mouse transplantation model was established (Fig. 6E), and mice were treated with clodronate liposomes to deplete macrophages in vivo. Clodronate treatment led to a significant reduction in the proportion of splenic F4/80^+^CD11b^+^ macrophages (Fig. 6F). Continuous in vitro fluorescence imaging revealed that macrophage depletion resulted in a marked decrease in tumor burden, accompanied by a reduction in the numbers of peritoneal metastatic lesions and the volume of ascites (Fig. 6G and Supplementary Fig. S6D).

Subsequently, the proliferative activity of tumor cells was verified in the macrophage-depleted mouse model. Experiments demonstrated that after macrophage depletion, the expression of Ki67 in tumor cells was downregulated. This result indicates that TAMs promote tumor growth, and tumor growth is inhibited when TAMs are depleted—consistent with the observations in the 3D bioprinting model (Fig. 6H). Given that macrophage-targeted therapy can delay the progression of ovarian cancer in mice, and platinum-based drugs serve as first-line chemotherapeutic agents for ovarian cancer, we further evaluated whether macrophage-targeted therapy could enhance the anti-tumor efficacy of chemotherapy for ovarian cancer. The results showed that, compared with the PBS control group and the single-drug treatment groups, the combined treatment with clodronate liposomes and cisplatin significantly inhibited the tumor growth rate (Fig. 6I).

The present study reveals that upon activation in tumor cells, IL7R promotes CXCL1 secretion by facilitating the nuclear translocation of NF-κB, which then binds to the CXCL1 promoter. Meanwhile, CXCL1 binds to CXCR2 on the surface of macrophages, inducing their polarization toward the immunosuppressive phenotype. These polarized macrophages, in turn, act on tumor cells by secreting cytokines such as IL-10, thereby promoting tumor cell proliferation and metastasis (Fig. 6J).

## Discussion

The IL-7/IL-7R axis has been well characterized in the context of immune development and hematologic cancers [40]. However, its function in solid tumors—especially ovarian cancer—remains largely undefined.

Cytokine-mediated regulation of downstream pathways may rely on the genetic profiles of tumor cells and their surrounding microenvironment [41]. The IL-7 signaling pathway is involved in normal lymphocyte development, peripheral T cell homeostasis, the regulation of immune tolerance, and mechanisms related to autoimmunity; it is also closely associated with the progression of various tumor types [30]. Notably, this pathway plays a critical role in shaping the tumor microenvironment [42]. Signals triggered by IL-7 are transduced to downstream target molecules through the JAK, PI3K, and MAPK pathways [40]. Under different internal and external conditions, distinct cell types may utilize unique pathways to mediate the function of IL-7R [43]. For instance, during the development of hematopoietic cells, IL-7 promotes the differentiation and survival of pro-B/pre-B-cell by activating the JAK-STAT pathway [44]. Studies have demonstrated that mice deficient in IL-7 exhibit arrest in B cell development [45]. In hematologic malignancies such as T-cell acute lymphoblastic leukemia (T-ALL), pharmacological inhibition of the JAK-STAT pathway induces cell death in mutant cells that express IL-7Rα [46].

The solid tumors and hematologic malignancies display distinct immune microenvironments. The immune microenvironment of solid tumors comprises tumor cells, immune cells, fibroblasts, and vascular endothelial cells, while that of hematopoietic malignancies is dominated by abnormal hematopoietic and immune cells, with a limited number of supportive stromal cells (e.g., fibroblasts) [47]. Notably, solid tumors exhibit context-dependent reprogramming of signaling pathways, which implies that differences in the microenvironmental may alter the preference for downstream IL-7R signal transduction [48]. Additionally, variations in the expression levels, conformation, and adapter proteins of IL-7R across different cell types might contribute to the selective activation of pathways. In non-small cell lung cancer (NSCLC), where the tumor microenvironment is primarily immunosuppressive, IL-7 induces the nuclear-to-cytoplasmic translocation of P53 in lung cancer cells. This translocation further regulates the AMPK/mTOR pathway and inhibits the autophagy of tumor cells [49, 50]. In primary prostate cancer, the immune microenvironment is characterized by suppressive myeloid cells and exhausted T cells, and the expression level of IL-7/IL-7R in this microenvironment is significantly higher than that in normal tissues. IL-7 stimulation can trigger the activation of the AKT and NF-κB pathways in prostate cancer cells by upregulating matrix metalloproteinases and epithelial-mesenchymal transition (EMT)-related proteins, thereby promoting the degradation of the extracellular matrix, the occurrence of EMT, and the metastatic potential of tumor cells [51, 52]. In ovarian cancer, the activation of NF-κB enhances the aggressiveness of tumor, facilitates the formation of an immune-evasive microenvironment, and recruits pro-tumorigenic immune cells, which in turn promote the survival and metastasis of cancer cells [53, 54]. This study demonstrated that IL-7R signaling is dependent on NF-κB signaling: NF-κB (RelA/p65) can directly bind to the promoter of CXCL1 (a critical downstream effector), whereas the traditionally recognized JAK/STAT and PI3K/AKT pathways remain unaffected by IL-7R knockout. These findings suggested that the signal transduction of IL-7R is regulated by the microenvironment.

Ovarian cancer is characterized by abnormal metabolic pathways, impaired antigen presentation, and enrichment of monocyte and immature macrophages. However, the dynamic interactions between the tumor microenvironment and cancer cells remain poorly understood [55]. This study demonstrated that IL-7R plays a pivotal role in remodeling the immune microenvironment of ovarian cancer, primarily by regulating the infiltration patterns of immune cells and the polarization of macrophages. Single-cell transcriptome sequencing revealed that IL-7R knockout enhanced the infiltration of mature B cells, CD8^+^ T cells, and macrophages into mice tumor tissues. Multiplex immunofluorescence staining confirmed that human ovarian cancer tissues with high IL-7R expression exhibited an “immune desert” phenotype, where immune cells were predominantly localized at the tumor periphery. Mechanistic studies indicated that IL-7R drives the polarization of TAM toward an immunosuppressive phenotype via the NF-κB-CXCL1-CXCR2 signaling axis. Integrated RNA-sequencing and proteomic analyses identified CXCL1 as a key regulatory molecule. While Spp1 (osteopontin) has been associated with tumor cell migration and invasion in lung and breast cancer, its function is primarily regarded as cell-autonomous, and no association with macrophage polarization has been reported [56, 57]. Although CXCL2 and CXCL3 belong to the CXC chemokine family, their roles in the polarization of macrophages in ovarian cancer remain undefined [58]. CXCL1 has been confirmed to regulate the formation of the tumor microenvironment and the behavior of cancer cells. In colorectal cancer, the CXCL1-CXCR2 axis promotes the secretion of VEGF and matrix metalloproteinases by macrophages via the activation of NF-κB and MAPK, thereby accelerating tumor angiogenesis and the degradation of the extracellular matrix [59]. In liver cancer, blocking this axis enhances the efficacy of doxorubicin-based chemotherapy by remodeling the tumor microenvironment [39]. Notably, previous studies have shown that CXCL1 promotes the proliferation of ovarian cancer cells via EGFR activation and mediates adiponectin-induced angiogenesis [60, 61]. This study further demonstrated that the expression of CXCL1 is regulated by IL-7R signaling: tumor cells with high IL-7R expression upregulate CXCL1 to drive the polarization of immunosuppressive macrophages. These findings provide a novel mechanism underlying the enrichment of immunosuppressive macrophages and the formation of an immunosuppressive microenvironment in ovarian cancer.

Intriguingly, recent studies have reported that in certain tumor types (e.g., hematologic malignancies), exogenous recombinant IL-7 (such as long-acting IL-7) combined with chimeric antigen receptor T (CAR-T) cell therapy suppresses tumor progression by enhancing T cell function [49, 62]. However, the present study documents an opposing effect in ovarian cancer: endogenous IL-7/IL-7R signaling promotes disease progression by driving tumor cell-autonomous proliferation and the formation of an immunosuppressive microenvironment. This discrepancy may stem from tumor-type-specific microenvironmental heterogeneity. In T cell-dominated tumors (e.g., lymphomas), IL-7 exerts anti-tumor effects by supporting T cell survival and expansion. In contrast, ovarian cancer is characterized as a “cold tumors”—a type characterized by macrophage infiltration and inherent immunosuppression [55]. In this context, tumor cells with high IL-7R expression may co-opt IL-7 signaling to promote the enrichment of immunosuppressive TAM via CXCL1-dependent mechanisms; this process counteracts the potential immunostimulatory effects of IL-7. Notably, the primary source of IL-7 in ovarian cancer may be the systemic circulation rather than the tumor microenvironment itself, which further amplifies the dominance of IL-7R-positive tumor cells in regulating the microenvironment.

Given the critical role of IL7R in driving the immunosuppressive tumor microenvironment and tumor progression, this study established a causal evidence chain linking IL7R to macrophage phenotypes through spatial correlation analysis in clinical samples, in vitro functional validation using human cell lines, and cross-species analysis of mechanistic conservation. Future studies will further validate these findings using patient-derived organoid-immune cell co-culture models or by sorting TAMs from patients’ tumor tissues for phenotypic analysis and functional assays, aiming to supplement direct causal evidence that more closely mimics clinical scenarios. From the perspective of clinical translation, our research supports the potential of targeting IL7R as a novel therapeutic strategy for ovarian cancer, particularly when combined with immunotherapies that modulate macrophages. Since IL7R signaling exerts its pro-tumor effects not only by promoting tumor cell proliferation and invasion but also by driving the polarization of immunosuppressive TAMs, combining IL7R inhibition with strategies that reprogram TAMs may enhance therapeutic efficacy. Currently, drugs such as CSF1R inhibitors or TLR agonists can shift TAMs from an immunosuppressive phenotype to an immunostimulatory one, reversing their immunosuppressive properties. In the future, combining these drugs with IL7R-targeted therapy could simultaneously block both the inducer of this pathway (IL7R-driven CXCL1) and its effector (suppressive TAMs). Additionally, stratifying patients based on IL7R expression to identify subgroups most likely to benefit would improve the precision of this therapeutic approach.

In this study, we elucidated the role of the IL-7/IL-7R axis in the ovarian cancer microenvironment and identified IL-7R expression in tumor cells as a critical target for the regulation of IL-7 signaling. The mechanisms mediated by IL-7R exert dual pro-tumor effects: promoting tumor cell proliferation and metastasis, as well as remodeling the immune microenvironment. These findings not only establish IL-7R as a driver of ovarian cancer progression but also provide new insights into how circulating IL-7 contributes to poor prognosis by facilitating tumor progression.

## Experimental section

### Clinical samples

Human patient samples were collected from the First Affiliated Hospital of Nanjing Medical University (Nanjing, China). The use of human specimens in this study was approved by the Ethics Committee of the First Affiliated Hospital of Nanjing Medical University (2023-SRFA-218), Nanjing, China, and written informed consent was obtained from all the patients or their legal guardians. The demographic data of the healthy donors and patients are summarized in Supplementary Table 1.

### Cell lines

The human embryonic lung cell line (MRC-5, RRID:CVCL_0440), human leukemia monocytic cell line (THP-1, RRID:CVCL_0006), mouse ovarian epithelial cancer cell line (ID-8, RRID:CVCL_IU14), human ovarian cancer cell lines (OVCAR3, RRID:CVCL_0465; A2780, RRID:CVCL_0134; HO-8910, CSTR:19375.09.3101HUMTCHu25; SKOV3, RRID:CVCL_0532), and human umbilical vein endothelial cells (HUVECs, 4201PAT-CCTCC00692) were maintained in our laboratory. The cells were cultured in high-glucose Dulbecco’s modified Eagle’s medium (H-DMEM, Bio-Channel, China) or Roswell Park Memorial Institute 1640 (RPMII, Bio-Channel, China) supplemented with 10% fetal bovine serum (FBS, Bio-Channel, China) and 1% penicillin/streptomycin at 37 °C in a 5% CO_2_ incubator. Generation of CRISPR/Cas 9 knockout cell lines: RNP complexes were prepared with guide RNA (gRNA) targeting mouse *Il7r* and Cas9 at a 5:1 to 10:1 ratio. Electroporation of the RNP complexes was performed using an electric transfection apparatus according to the manufacturer’s instructions for the Lonza and Thermo Fisher Scientific Neon Transfection Systems. Single-cell isolation and expansion were performed using a DispenCell single-cell dispenser (Molecular Devices). Generation of stable cell lines: Lentiviral vectors containing IL7R-shRNA and shLuc control vectors were purchased from WZ Biosciences Inc. The target sequences for IL7R shRNA were as follows: Sequence1: GGAGGTAAAGTGCCTGAATTTCTCGAGAAATTCAGGCACTTTACCTCCTTTTTT; Sequence2: CTGATTGGAAAGAGCAATATATTCAAGAGATATATTGCTCTTTCCAATCAGTTTTTT. The lentivirus was added to the cultured cells and incubated for 48 h. Infected cells were selected using purinomycin (HY-K1057, MCE, China) for 1 week. In this study, phorbol 12-myristate 13-acetate (PMA, 30 ng/mL; HY-18739, MCE, China) was used to induce differentiation of THP-1 cells into macrophages. The culture medium was replaced three times a week, and the cells were passaged when the confluence exceeded 80%. All cell lines were regularly tested for mycoplasma contamination during the experiment, and all results were negative.

### Mice

All the mice were bred and maintained under specific pathogen-free (SPF) conditions. Littermate mice or appropriate age- and sex-matched controls were used for analysis. Female C57BL/6 mice aged 6–8 weeks were used in all experiments.

Establishment of abdominal metastasis and subcutaneous tumor models for immunocompetent C57BL/6 mice, *Il7r*-WT and *Il7r*-KO cells in the logarithmic growth phase were inoculated into the abdominal cavity at 5×10^6^ cells per mouse and into the left axilla at 1 × 10^6^ cells per mouse, respectively.

Establishment of macrophage depletion models and combination therapy models: Based on the peritoneal metastasis model, F4/80+CD11b+ macrophages were depleted using clodronate liposomes, and cisplatin chemotherapy was administered via intraperitoneal injection.

All animal experiments were performed in accordance with protocols approved by the Institutional Animal Care and Use Committee of Nanjing Medical University (Animal protocol number: IACUC-2406112).

### Cell function experiment in vitro

Colony formation assay: WT and KO cells (500 cells per pore) were seeded into a 6-well plate (LABSELECT, China) and cultured at 37 °C for 9 days. The medium was changed every 2 days. Images were captured using a CKX53 microscope (OLYMPUS, Japan). Transwell migration assay: Cell migration was assessed using Transwell polycarbonate membranes (Corning, USA) with an 8-µm pore size to separate the upper and lower chambers. WT and KO cells (5 × 10^4^ cells) were seeded into the upper chamber with 200 µL of serum-free medium, and 600 µL of medium containing 10% FBS was added to the lower chamber. The Transwell system was incubated at 37 °C in 5% CO₂ for 24 h. Migrating cells were stained with crystal violet (BL802A, Biosharp, China) and counted in five randomly selected fields under a CKX53 microscope (OLYMPUS, Japan). Scratch wound healing assay: WT and KO cells were seeded in 6-well plates (LABSELECT, China) at 1 × 10^6^ cells per well. Once the cell confluence exceeded 95%, the monolayers were washed with PBS, and a scratch was created using a sterile p200 pipette tip. Wound closure was imaged every 12 h for 24 h using a CKX53 microscope (OLYMPUS, Japan).

### Flow cytometry

Cells were stained with the following flow cytometry antibodies and analyzed using a BD FACSCanto™ Plus flow cytometer (BD Biosciences). Briefly, unlabeled Fc receptor-blocking antibodies were used to block nonspecific binding, followed by the addition of live/dead cell viability dyes to exclude dead cells. The cell surface targets were stained. The data were analyzed using FlowJo software (Tree Star). The flow cytometry antibodies used are listed in Table 1.

**Table 1:** The names of the antibodies used in flow cytometry, their manufacturing companies, and catalog numbers.

Reagent or resource	Source	Identifier
APC anti-mouse F4/80	Biolegend	Cat# 123115
FITC anti-mouse/human CD11b	Biolegend	Cat# 101205
PE anti-mouse CD206(MMR)	Biolegend	Cat# 141705
PE/Cyanine7 anti-mouse I-A/I-E	Biolegend	Cat# 107629
FITC anti-human CD80	Biolegend	Cat# 375405
APC anti-human CD45	Biolegend	Cat# 304012
PE Anti-Human CD206 (15-2)	PTG	Cat# PE-65155
PE/Cyanine7 anti-human CD11b	Biolegend	Cat# 301321
Human TruStain FcXTM	Biolegend	Cat# 422301
(Fc Receptor Blocking Solution)		
Anti-Mouse CD16/32 (93)	PTG	Cat# 65057-1-Ig
Zombie Aqua™ Fixable Viability Kit	Biolegend	Cat# 423101
PE Anti-Mouse CD45	PTG	Cat# PE-65087

### Quantitative real-time polymerase chain reaction

Quantitative real-time PCR (qPCR) and data analysis were performed on a LightCycler 480 II (Roche, Switzerland). β-actin served as an internal control. The forward and reverse primers that were used are listed in Table 2.

**Table 2:** PCR primer sequences.

Species	Gene	Forward sequence	Reverse sequence
Mouse	Il-1β	TGGACCTTCCAGG ATGAGGACA	GTTCATCTCGGAG CCTGTAGTG
Mouse	Il-6	TACCACTTCACAA GTCGGAGGC	CTGCAAGTGCATC ATCGTTGTTC
Mouse	Il-10	CGGGAAGACAATA ACTGCACCC	CGGTTAGCAGTAT GTTGTCCAGC
Mouse	Tgf-β	TGATACGCCTGAG TGGCTGTCT	CACAAGAGCAGTG AGCGCTGAA
Mouse	iNOS	GAGACAGGGAAGT CTGAAGCAC	CCAGCAGTAGTTG CTCCTCTTC
Mouse	Arg-1	CATTGGCTTGCGAGACGTAGAC	GCTGAAGGTCTCT TCCATCACC
Mouse	Tnf-α	GGTGCCTATGTCT CAGCCTCTT	GCCATAGAACTGA TGAGAGGGAG
Mouse	Il-12	TTGAACTGGCGTT GGAAGCACG	CCACCTGTGAGTT CTTCAAAGGC
Mouse	Cxcl1	TCCAGAGCTTGAA GGTGTTGCC	AACCAAGGGAGCTT CAGGGTCA
Mouse	Cxcl2	CATCCAGAGCTTG AGTGTGACG	GGCTTCAGGGTCA AGGCAAACT
Mouse	S100a6	GCAAGGAAGGTGA CAAGCACAC	CCTGATCCTTGTT ACGGTCCAG
Human	β-actin	CATTGCTGACAGG ATGCAGAAGG	TGCTGGAAGGTGG ACAGTGAGG
Human	IL-7R	ATCGCAGCACTCA CTGACCTGT	TCAGGCACTTTAC CTCCACGAG

### Western blotting

Cell lysates were prepared in RIPA lysis buffer (P0013C, Beyotime, China) at 4 °C and separated by SDS-PAGE using precast gels from ACE Biotechnology. The proteins were then transferred onto a PVDF membrane (ISEQ00010, Millipore, Germany). After blocking with 5% nonfat dry milk, the membranes were probed with primary antibodies.

### Bone marrow-derived macrophage (BMDM) cultured

Bone marrow cells were isolated from the femurs of C57BL/6 mice. Red blood cells were removed using red blood cell lysis buffer (00-4333-57, Invitrogen, USA). Total bone marrow cells were suspended in high-glucose Dulbecco’s modified Eagle’s medium (H-DMEM, Bio-Channel, China) supplemented with 10% FBS and 20 ng/mL GM-CSF (RP01206, ABclonal, China), and seeded into a 6-well plate (LABSELECT, China).

### Co-culture assays

BMDMs (5 × 10^5^ cells per pore) were co-cultured with tumor cells or gradient concentrations of human recombinant IL-7 for 48 h to prepare them for subsequent experiments.

### Immunofluorescence analysis

The cells were seeded onto cell culture slides (801007, Nest, China) and fixed with 4% paraformaldehyde (BL539A, Biosharp, China) for 10 min. The cells were then permeabilized with 0.1% Triton X-100 (P0096-100ml, Beyotime, China) at room temperature for 10 min, followed by blocking with blocking buffer (P0260, Beyotime, China) for 10 min. The cells were then incubated with primary antibody (A19653, ABclonal, China) overnight at 4 °C, washed, and incubated with goat anti-rabbit IgG (SA00013-2, ProteinTech, China) at 37 °C for 1 h. Nuclei were counterstained with DAPI (0100-20, SouthernBiotech, USA), and the slides were visualized under an inverted fluorescence microscope (ECLIPSE Ti, Nikon, Japan). Paraffin sections were dewaxed in xylene, dehydrated through ethanol gradients, and subjected to antigen retrieval using a microwave in EDTA antigen repair buffer. The remaining steps are as follows. The primary and secondary antibodies used for immunofluorescence are listed in Table 3.

**Table 3:** Names, manufacturing companies, and catalog numbers of the antibodies used in Western Blot and multicolor immunofluorescence.

Reagent or resource	Source	Identifier
pan Cytokeratin	PTG	Cat# 26411-1-AP
Ki67	ABclonal	Cat# A20018
IL7R Antibody	Affbiotech	Cat# DF6362
CD68	PTG	Cat# 66231-2-Ig
β-actin	ABclonal	Cat# A2235
CD206	ABclonal	Cat# A21014
pan Cytokeratin	HUABIO	Cat# HA601138
CXCL1	PTG	Cat# 12335-1-AP
GAPDH	PTG	Cat# 10494-1-AP
IL-7	Invitrogen	Cat# PA5-119844
CD19	PTG	Cat# 27949-1-AP
CD86	PTG	Cat# 26903-1-AP
CD8	PTG	Cat# 66868-1-Ig

### Immunohistochemistry staining

Immunohistochemistry (IHC) staining was performed as previously described [63]. Briefly, the sections were processed, deparaffinized, rehydrated, and incubated with primary antibodies overnight at 4 °C, followed by incubation with HRP-conjugated secondary antibodies at 37 °C for 1 h. Staining was visualized by incubation with 3, 3′-diaminobenzidine substration, which resulted in a brown-colored precipitate at the antigen site. Nuclei were counterstained with hematoxylin.

### Bioluminescence imaging

The mice were anesthetized with 1.0–1.5% isoflurane. D-Luciferin potassium salt (ST198, Beyotime, China) was injected intraperitoneally at a dose of 150 mg/kg. After 10 min, according to the manufacturer’s instructions, bioluminescent signals from the entire animal were detected using an IVIS Spectrum Imaging System (PerkinElmer).

### RNA-seq and data analysis

Total RNA was extracted using TRIzol® reagent (Invitrogen). Sequencing libraries were generated with Novogene using the NEB Next Ultra RNA Library Prep Kit for Illumina, followed by sequencing on an Illumina NovaSeq sequencer. For RNA-seq data analysis, the R package pheatmap was used to generate gene expression heatmaps, and differentially expressed genes (DEGs) were identified using the DeSeq2 package in R. Gene Set Enrichment Analysis was performed for functional enrichment analysis of DEGs.

### 3D-bioprinting

The concentric cylinder consists of four layers, with five inner layers and four outer layers, and a final height of 1.0 mm. SEM Imaging: Photocured porous gelatin methacryloyl (GelMA, EFL-GM-PR-001, EFL) was fixed with 2% paraformaldehyde at room temperature for 15 min. After lyophilization, the sample was mounted on a stub, and the surface of each cross-section was coated with a thin layer of gold using a sputter coater. The microstructure and pore size of each sample were observed by scanning electron microscopy (SEM, Hitachi Regulus8100). Young’s Modulus Measurement: Using a CMT6103 mechanical test system (MTS\SANS), unconfined compression was applied to the samples at a strain rate of 1 mm/min until a total strain of 50% was reached. Construction of 3D Bioprinted Tissues: A dual-nozzle 3D bioprinter (SunP BioMaker 2i, SUNP BIOTECH) was used to fabricate tumor tissues. A 7% (w/v) porous GelMA cell suspension was then prepared. Tissues were fabricated via layer-by-layer extrusion, collected in a 12-well plate (LABSELECT), and immediately crosslinked under a 405 nm light source positioned close to the plate to solidify the hydrogel. Specifically, in Video 1, we demonstrate a 3D bioprinter utilizing a single nozzle (a photocurable nozzle) to print a 1 × 1 × 1 cm cubic structure for elastic modulus testing. The nozzle prints in a top-to-bottom and left-to-right direction, forming dense strips. After each layer is printed, the 3D bioprinter automatically activates a UV lamp to cure the current structure. In Video 2, we show the 3D bioprinter constructing a tumor-stroma model: after Nozzle 1 (a thermosensitive nozzle) completes the printing of the tumor core, the bioprinter automatically switches to Nozzle 2 (a photocurable nozzle) to print the stroma. Similarly, after each layer is printed, the 3D bioprinter automatically turns on the UV lamp to cure the current structure.

### CUT&Tag Assay

The Hyperactive Universal CUT&Tag Assay Kit for Illumina Pro (TD904-01 Vazyme, China) was used according to the manufacturer’s protocol [64]. Briefly, 1 × 10^5^ cells were used per sample. For sequencing library construction, the TruePrep Index Kit V2 for Illumina (TD202 Vazyme, China) was used for DNA PCR amplification. For ID-8 cells, the number of PCR cycles was set to 14.

### Single-cell RNA sequencing and data analysis

Freshly isolated tissues were digested into single cells using tissue dissociation solution (Singleron, China). Single-cell suspensions (2 × 10^5^ cells/mL) in PBS (HyClone, USA) were loaded onto a microwell chip using the Singleron Matrix Single Cell Processing System. Barcoding beads were subsequently collected from the microwell chip, followed by reverse transcription of the captured mRNA to generate cDNA for PCR amplification. The amplified cDNA was then fragmented and ligated using sequencing adapters. scRNA-seq libraries were constructed according to the GEXSCOPE® Single Cell RNA Library Kit (Singleron; Ref) protocol. Individual libraries were diluted to 4 nM, pooled, and sequenced on an Illumina NovaSeq 6000 with 150 bp paired-end reads. Raw reads were processed to generate gene expression profiles using CeleScope v1.5.2 (Singleron Biotechnologies, China) with default parameters. Briefly, barcodes and UMIs were extracted from the R1 reads and corrected for errors. Adapter sequences and poly(A) tails were trimmed from R2 reads, and the trimmed R2 reads were aligned against the mm10 transcriptome using STAR (v2.6.1b). Uniquely mapped reads were assigned to exons using FeatureCounts (v2.0.1). Reads successfully assigned to the same cell barcode, UMI, and gene were grouped to generate the gene expression matrix for downstream analysis.

### Statistical analysis

All statistical analyses were performed using GraphPad Prism v9.5. All experiments were performed in triplicate unless otherwise noted. For comparisons between two groups, an unpaired Student’s t-test was used. Significant differences across multiple groups were evaluated using one-way analysis of variance (ANOVA), followed by Tukey’s post-hoc test for parametric data or the Kruskal-Wallis test for non-parametric data. Statistical significance was defined as P < 0.05, with the notation: P < 0.05 (*), P < 0.01 (**), P < 0.001 (***), P < 0.0001 (****), and non-significant (ns, *P* > 0.05).

## Supplementary information

Supplementary Figure Legend

Video 1

Video 2

Original Western Blots

Supplementary Figure 1

Supplementary Figure 2

Supplementary Figure 3

Supplementary Figure 4

Supplementary Figure 5

Supplementary Figure 6

Supplementary Table S1

Supplementary Table S2

Supplementary Table S3

Supplementary Table S4

Supplementary Table S5

## Data Availability

The raw sequence data reported in this paper have been deposited in the Genome Sequence Archive (Genomics, Proteomics & Bioinformatics 2021) in National Genomics Data Center (Nucleic Acids Res 2022) [65, 66], China National Center for Bioinformation/Beijing Institute of Genomics, Chinese Academy of Sciences (GSA: CRA026211, CRA026236, CRA026188) that are publicly accessible at https://ngdc.cncb.ac.cn/gsa. The datasets analyzed by CIBERSORT were obtained from TCGA. No original code was generated in this study.
