# Supplementary material for: IL7R remodels immunosuppression tumor microenvironment and promotes macrophage polarization by regulating NF-κB/CXCL1 axis in ovarian cancer
Source: Cell Death Dis. 2025 Dec 8;17(1):95. doi: 10.1038/s41419-025-08312-6 (PMC12830660; doi:10.1038/s41419-025-08312-6)
Supplement: Supplementary file 1 — Supplementary Figure Legend [file 41419_2025_8312_MOESM1_ESM.doc]

**IL7R Remodels Immunosuppression Tumor Microenvironment and Promotes Macrophage Polarization by Regulating NF-κB/CXCL1 Axis in Ovarian Cancer**

Yeqing Zheng*, Cheng Qian*, Shuyi Zhang, Meichen Wen, Xun Xu, Xuan Zhou, Yicong Wan, Lin Zhang^#^, Wenjun Cheng^#^

**Supplement Figure 1: IL7R expression and functional validation in ovarian cancer models.**

(A) ELISA was used to measure the concentrations of IL-7 in plasma and ascites from patients with ovarian cancer (N=11) and those with benign ovarian tumors (N=7); (B) Western blot analysis confirming efficient IL7R knockout at the protein level in ID-8 cells. β-actin served as a loading control; (C) Sanger sequencing chromatograms demonstrating genomic editing efficiency at the IL7R locus, showing indels at the sgRNA-targeted site; (D) Macroscopic view of subcutaneous tumor formation in the left axilla of mice (N=5). A representative image depicts tumor size at the experimental endpoint (day 28 post-injection); (E) Representative images from the peritoneal metastasis model: Left: Comparative ascites volume in mice injected with Il7r-KO versus WT cell groups; Right: Diaphragmatic metastatic foci, with white arrows indicating tumor nodules. Statistical significance: ns (not significant), *P < 0.05, **P < 0.01, ***P < 0.001, ****P < 0.0001. Data are presented as mean ± SEM.

**Supplement Figure 2: 3D bioprinted tumor-stroma coculture model and IL7R functional analysis.**

(A) Colony formation assay showing reduced proliferative capacity of *Il7r*-KO ID8 cells compared with WT controls, visualized by crystal violet staining; (B) Transwell migration assay demonstrating decreased cell motility in *Il7r*-KO cells after 24 hours of incubation. Scale bar: 100 μm; (C) Wound healing assay confirming reduced cell migration in *Il7r*-KO cells, with representative images captured at 0, 12, and 24 hours. Scale bar: 50, 200 μm; (D) Colony formation assay with exogenous IL-7 treatment in WT and KO cells. IL-7 partially rescued proliferation in WT but not KO cells; (E) Wound healing assay with IL-7 treatment in WT and KO cells. IL-7 promoted migration specifically in WT cells. Scale bar: 100 μm; (F) Transwell migration assay of *Il7r*-KO and WT cells treated with IL-7 for 24 hours. Scale bar: 50 μm; (G) 3D-printed cubic hydrogel model (10×10×10 mm) used for mechanical property testing; (H) 3D concentric circle model (8×8×1 mm) simulating the tumor-stroma interface, with live/dead staining (calcein-AM for live cells, propidium iodide for dead cells) at days 1, 4, and 7. Scale bars: 100 μm.

**Supplement Figure 3：Single-cell and spatial analysis of IL7R-associated tumor-immune interactions in ovarian cancer.**

(A) UMAP projection of single-cell RNA sequencing (scRNA-seq) data from murine ovarian cancer tissues, color-coded by annotated cell type clusters; (B) Dot plot depicting cluster-specific marker genes. Dot size represents the percentage of cells expressing each gene, and color intensity indicates log-normalized mean expression levels; (C) Correlation analysis (GEPIA database) of IL7R expression with immune cell markers (CD68, CD206, CD19, CD8) in human ovarian cancer (TCGA-OV dataset). Pearson’s correlation coefficients (r) and p-values are indicated; (D, E) Volcano plot of differentially expressed genes in B cells and T cells (data from scRNA-seq); (F) Heatmap of co-expression patterns between IL7R and immune markers (CD68, CD206, CD19, CD8) based on quantitative immunofluorescence scoring of tissue microarray (TMA) sections; (G) Kaplan-Meier survival analysis of progression-free survival (PFS) in ovarian cancer patients stratified by IL7R expression (high vs. low; data from KM-Plotter).

**Supplement Figure 4：IL7R modulates macrophage polarization and migration in ovarian cancer.**

(A) Flow cytometry analysis of bone marrow-derived macrophages (BMDMs) treated with recombinant IL-7 (0-80 ng/mL): Representative flow cytometry plots showing CD206^+^ (anti-inflammatory-polarized) and MHC II^+^ (Pro-inflammatory-polarized) populations; quantification of anti-inflammatory/pro-inflammatory polarization shift; (B) qPCR validation of macrophage polarization marker genes (pro-inflammatory: iNOS, IL-1β, Tnf-α; anti-inflammatory: Arg1, Il-10, Tgf-β) in IL-7-treated BMDMs; (C) *Il7r*-dependent macrophage polarization in co-culture systems: qPCR analysis of macrophage marker genes in BMDMs co-cultured with *Il7r*-KO or WT tumor cells, with or without recombinant IL-7 (10 ng/mL). Results demonstrate that IL7-mediated macrophage reprogramming requires tumor cell-derived IL7R; (D) THP-1-derived macrophage polarization in response to IL-7 stimulation. THP-1 cells were primed with PMA (20 ng/mL) for 24 hours to generate M0 macrophages, then treated with IL-7 (0-80 ng/mL) for 48 hours; (E) KEGG pathway enrichment analysis of differentially expressed genes (DEGs) was performed using scRNA-seq data from *Il7r*-WT and *Il7r*-KO murine ovarian cancer tissues; (F) Flow cytometry gating strategy for macrophage analysis: Gated on live cells (FSC-A/SSC-A doublet exclusion); Selected CD11b^+^F4/80^+^ cells as macrophages; Analyzed anti-inflammatory polarization via CD206 expression.

**Supplement Figure 5: Multi-omics analysis reveals *Il7r*-KO-driven transcriptional and proteomic reprogramming via NF-κB/CXCL1 axis.**

(A) Correlation analysis of IL7R expression with CXCL1/CXCL2/CXCL3 in human ovarian cancer tissues. Data from the TISIDB database were used to assess Spearman’s rank correlation between IL7R and CXCL chemokines (CXCL1, CXCL2, CXCL3) in TCGA-OV cohort; (B) Validation of RNA-seq identified DEGs by quantitative PCR (qPCR); (C) Immunohistochemical staining of CXCL2 in murine tumor tissues. Images were captured at ×100 (scale bar=200 μm) and ×1000 (scale bar=20 μm) magnification; positive staining (brown) was quantified in tumor epithelial cells; (D) Transcription factor (TF) prediction for CXCL1 gene regulation. Overlapping TFs were visualized in a Venn diagram; (E) Flow cytometry analysis of immunosuppressive macrophage polarization in THP-1 co-cultured with sh-IL7R OVCAR3 cells treated with or without CXCL1 and sh-NC OVCAR3 tumor cells (N=3).

**Supplement Figure 6: NF-κB-dependent regulation of macrophage polarization and migration by ovarian cancer cells, and therapeutic effects of macrophage depletion.**

(A) Flow cytometry analysis of macrophage polarization in BMDMs co-cultured with murine ovarian cancer cells treated with CXCR2 inhibitor. Representative FACS plots and quantification of polarization ratios are shown; (B) qPCR validation of macrophage polarization in BMDMs co-cultured with murine ovarian cancer cells treated with CXCR2 inhibitor; (C) Transwell migration assay evaluating macrophage polarization in BMDMs co-cultured with murine ovarian cancer cells treated with CXCR2 inhibitor. Representative images (scale bar: 100 µm) and quantification are shown; (D) Macroscopic assessment of metastatic burden in the peritoneal dissemination model after clodronate liposome treatment. Representative images show ascites volume (left) and diaphragmatic metastatic foci (right, white arrows indicate tumor nodules) in mice treated with clodronate liposomes or PBS control.
