# Supplementary figures and images for: IL7R remodels immunosuppression tumor microenvironment and promotes macrophage polarization by regulating NF-κB/CXCL1 axis in ovarian cancer
Source: Cell Death Dis. 2025 Dec 8;17(1):95. doi: 10.1038/s41419-025-08312-6 (PMC12830660; doi:10.1038/s41419-025-08312-6)

Figure 1D

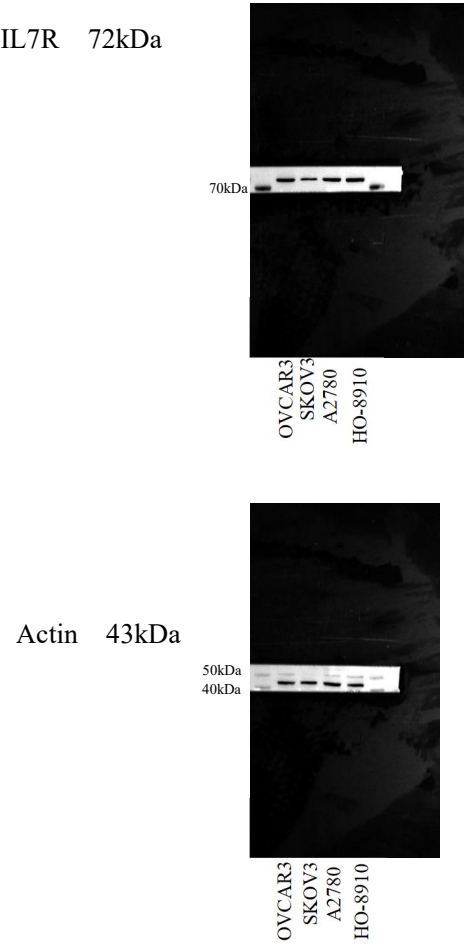

Figure 2F

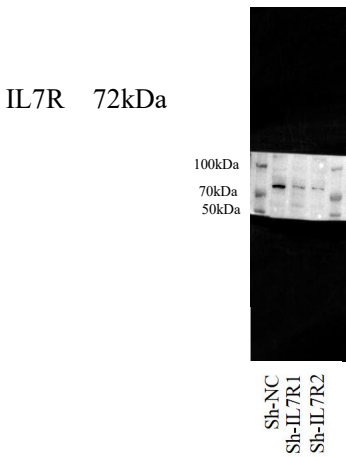

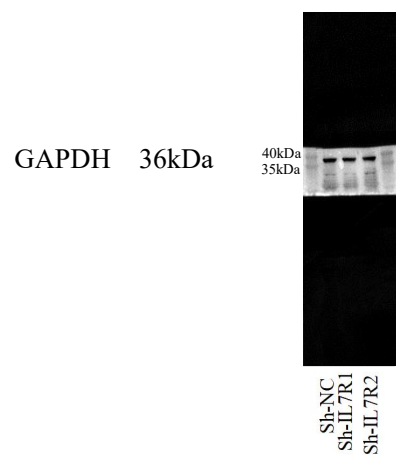

Figure 5H

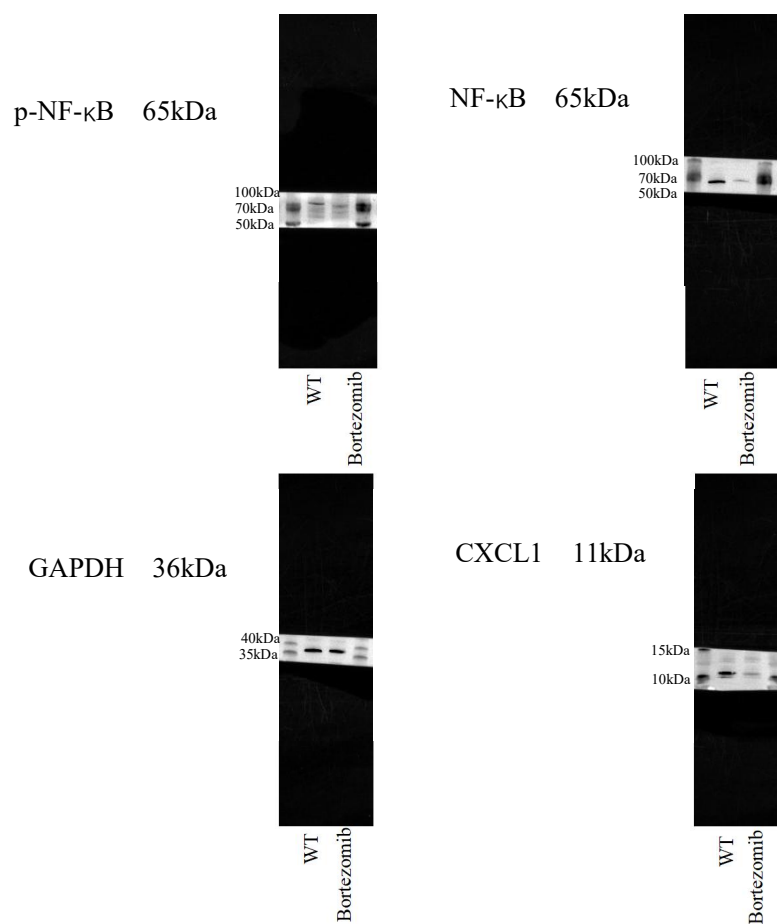

Figure 5I

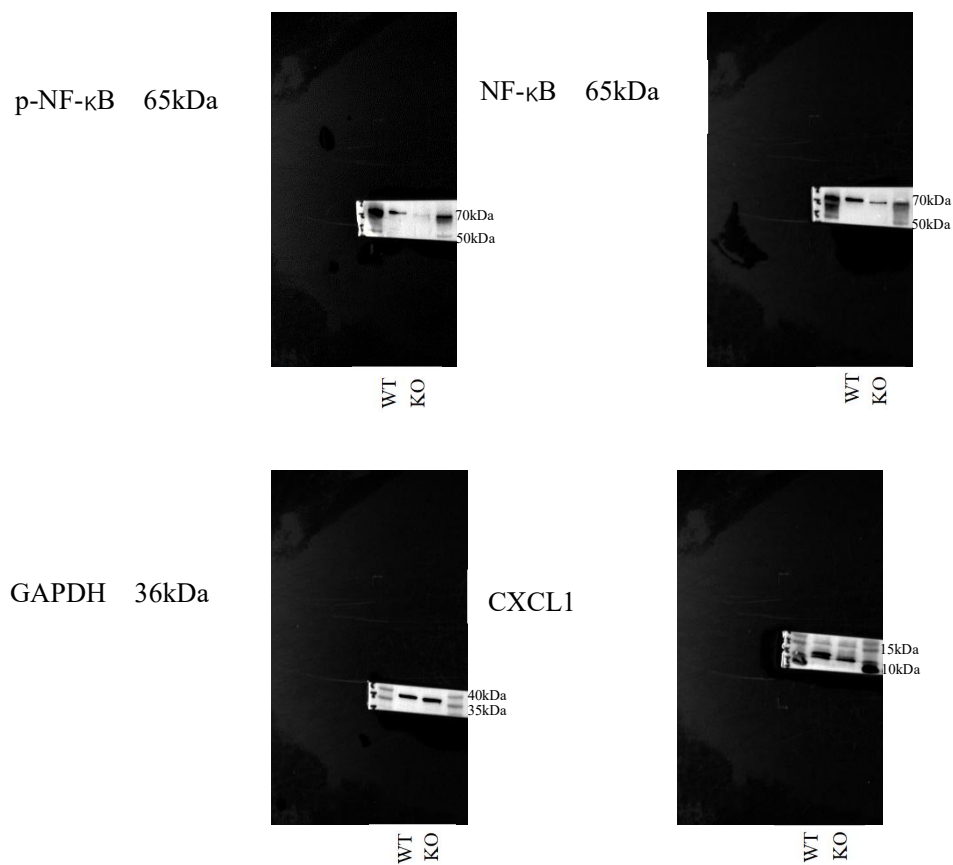

**Supplement Figure 1A**

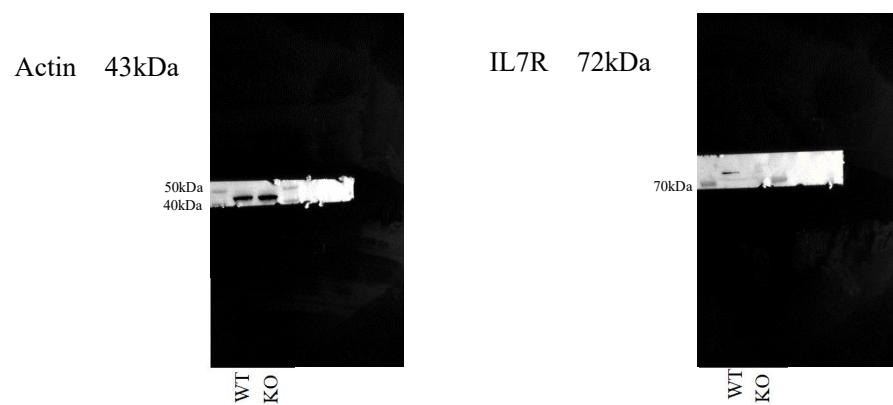

Supplement: Supplementary file 4 — Original Western Blots [file 41419_2025_8312_MOESM4_ESM.pdf]

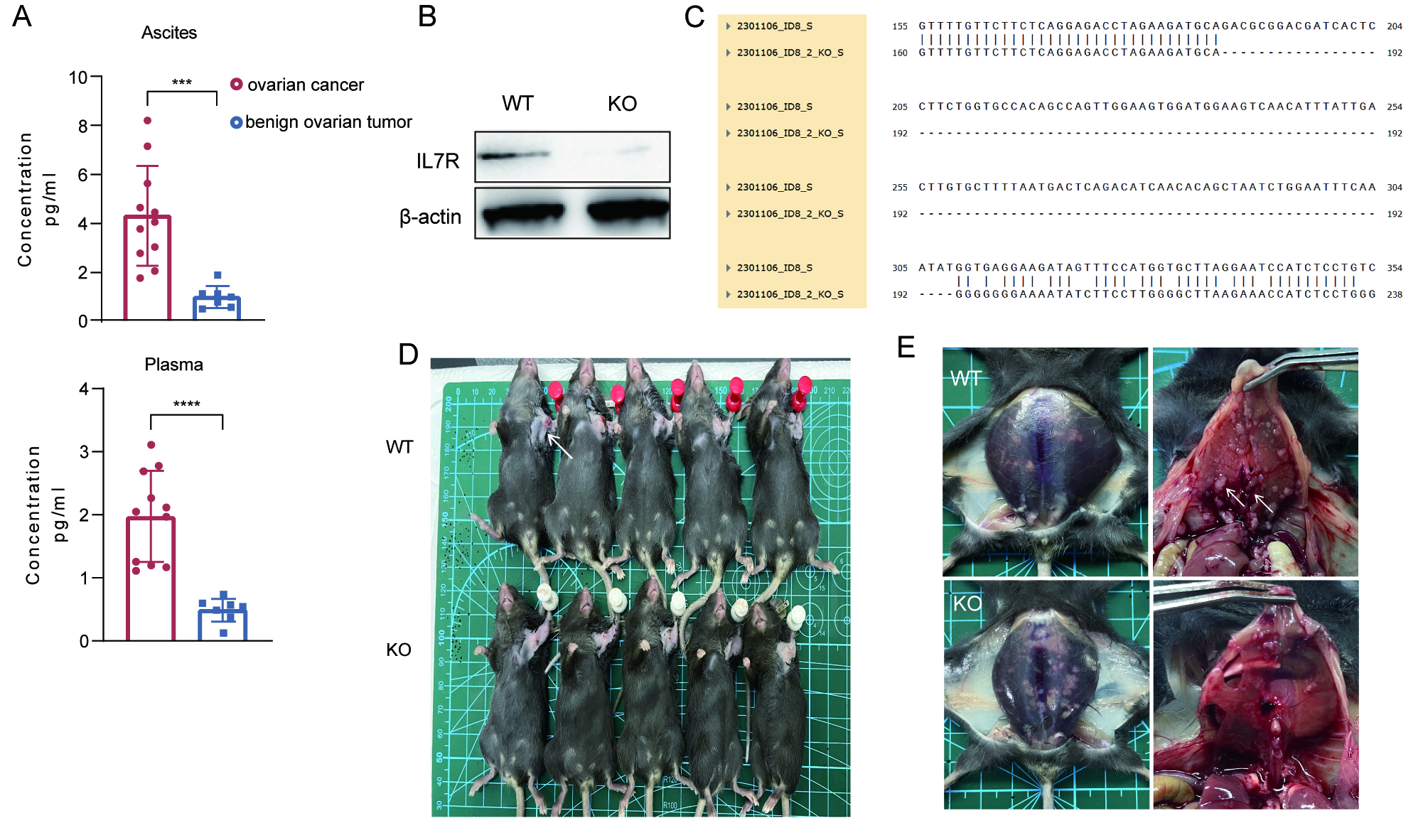

Supplement: Supplementary file 5 — Supplementary Figure 1 [file 41419_2025_8312_MOESM5_ESM.tif]

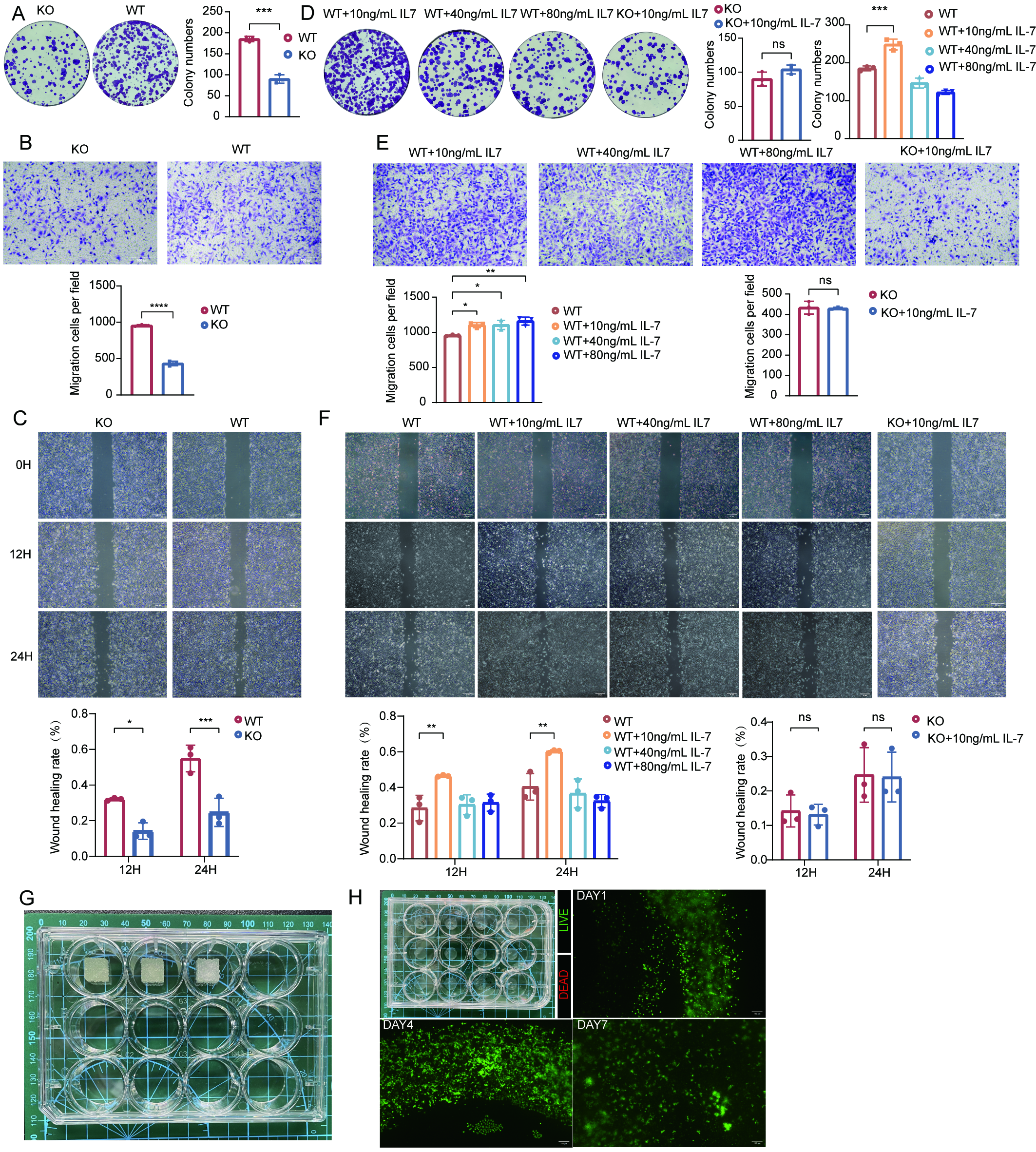

Supplement: Supplementary file 6 — Supplementary Figure 2 [file 41419_2025_8312_MOESM6_ESM.tif]

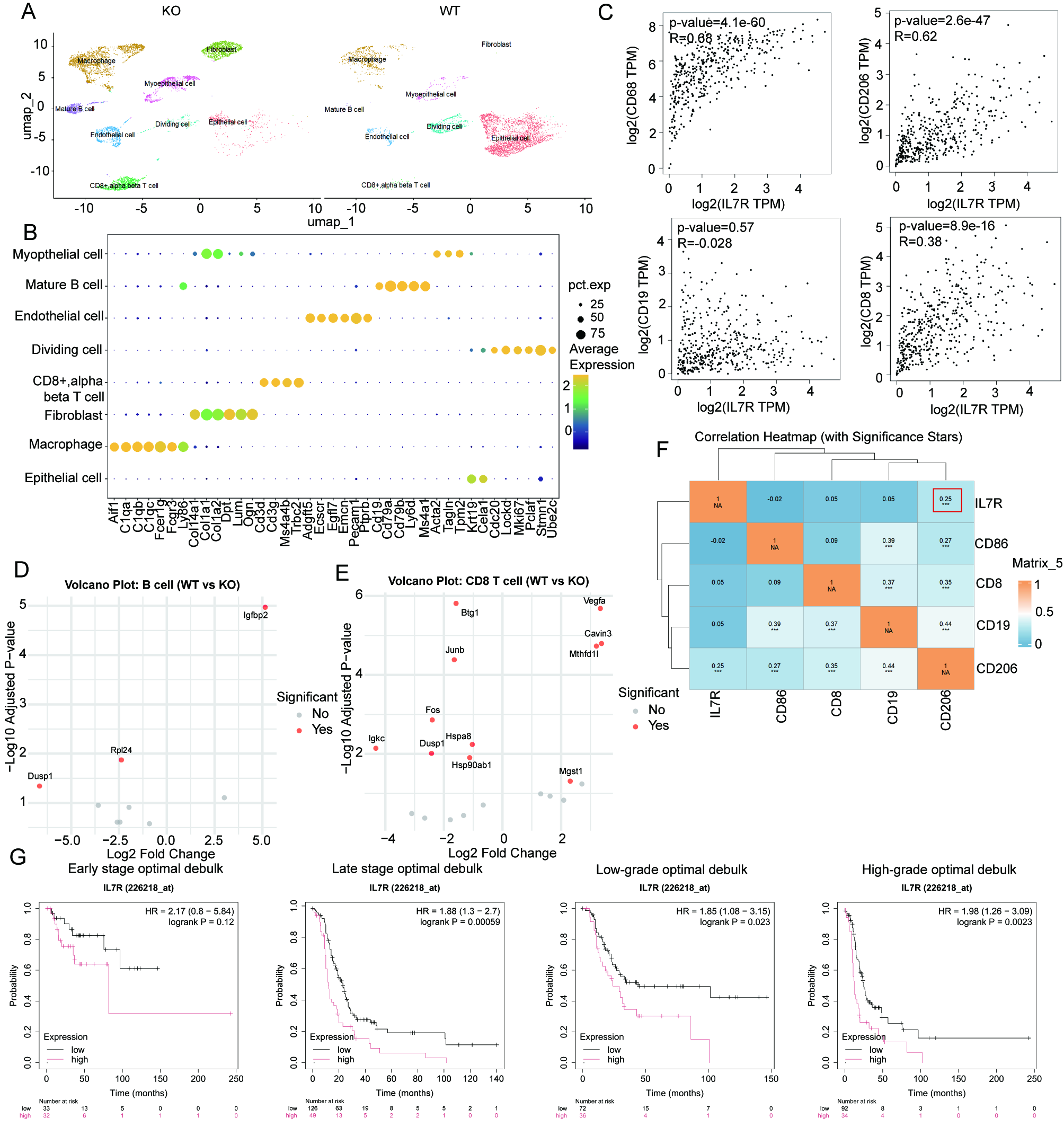

Supplement: Supplementary file 7 — Supplementary Figure 3 [file 41419_2025_8312_MOESM7_ESM.tif]

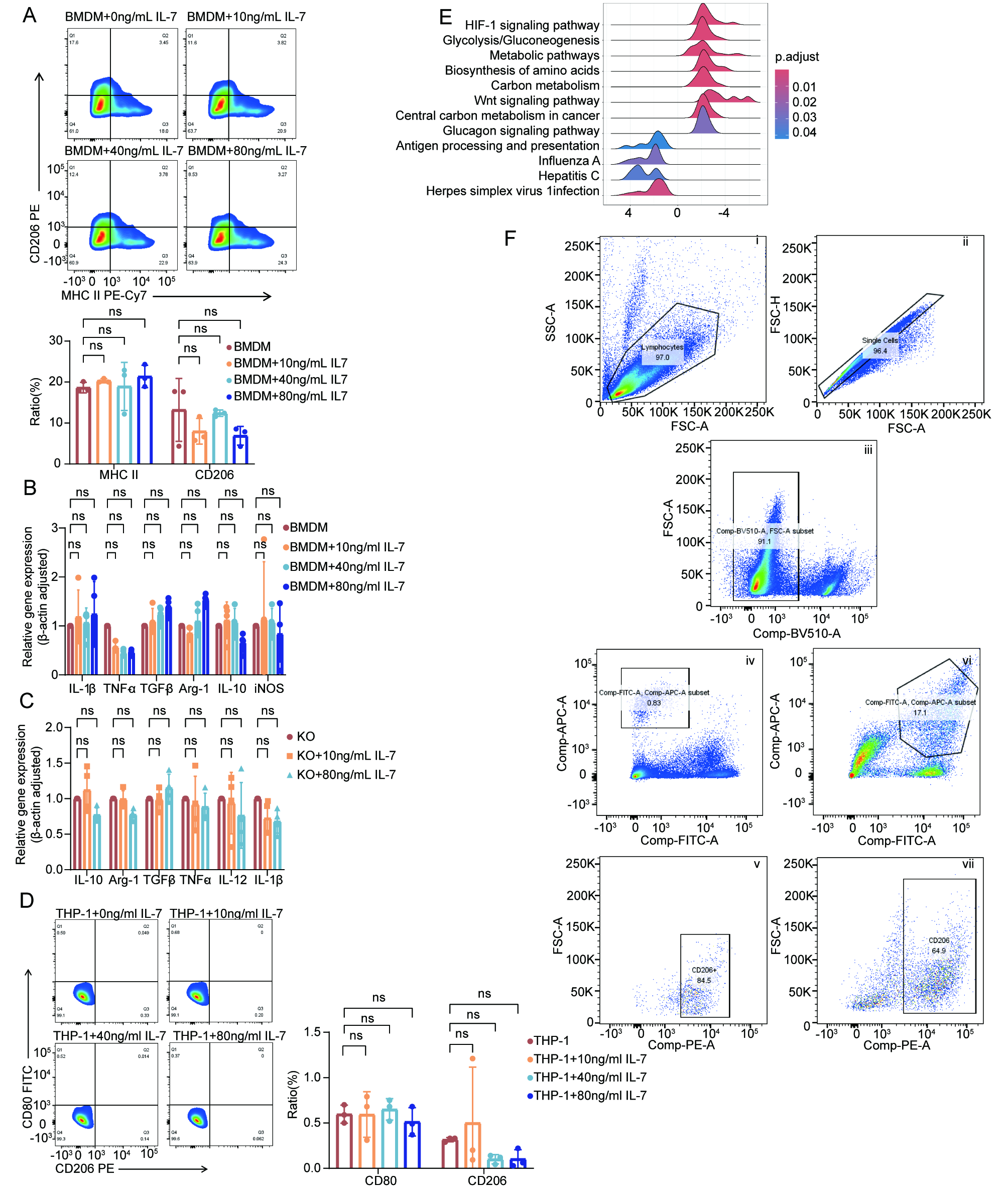

Supplement: Supplementary file 8 — Supplementary Figure 4 [file 41419_2025_8312_MOESM8_ESM.tif]

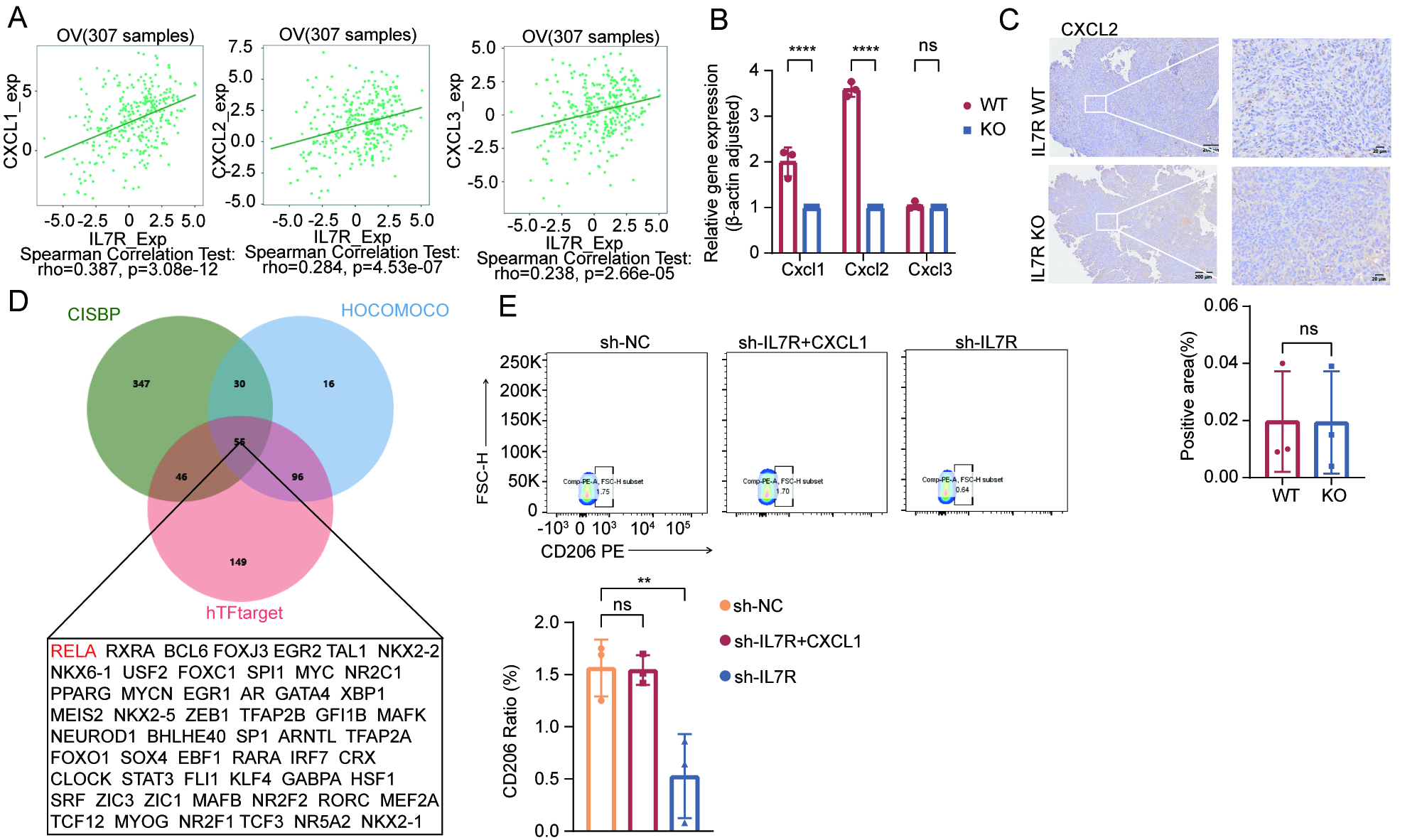

Supplement: Supplementary file 9 — Supplementary Figure 5 [file 41419_2025_8312_MOESM9_ESM.tif]

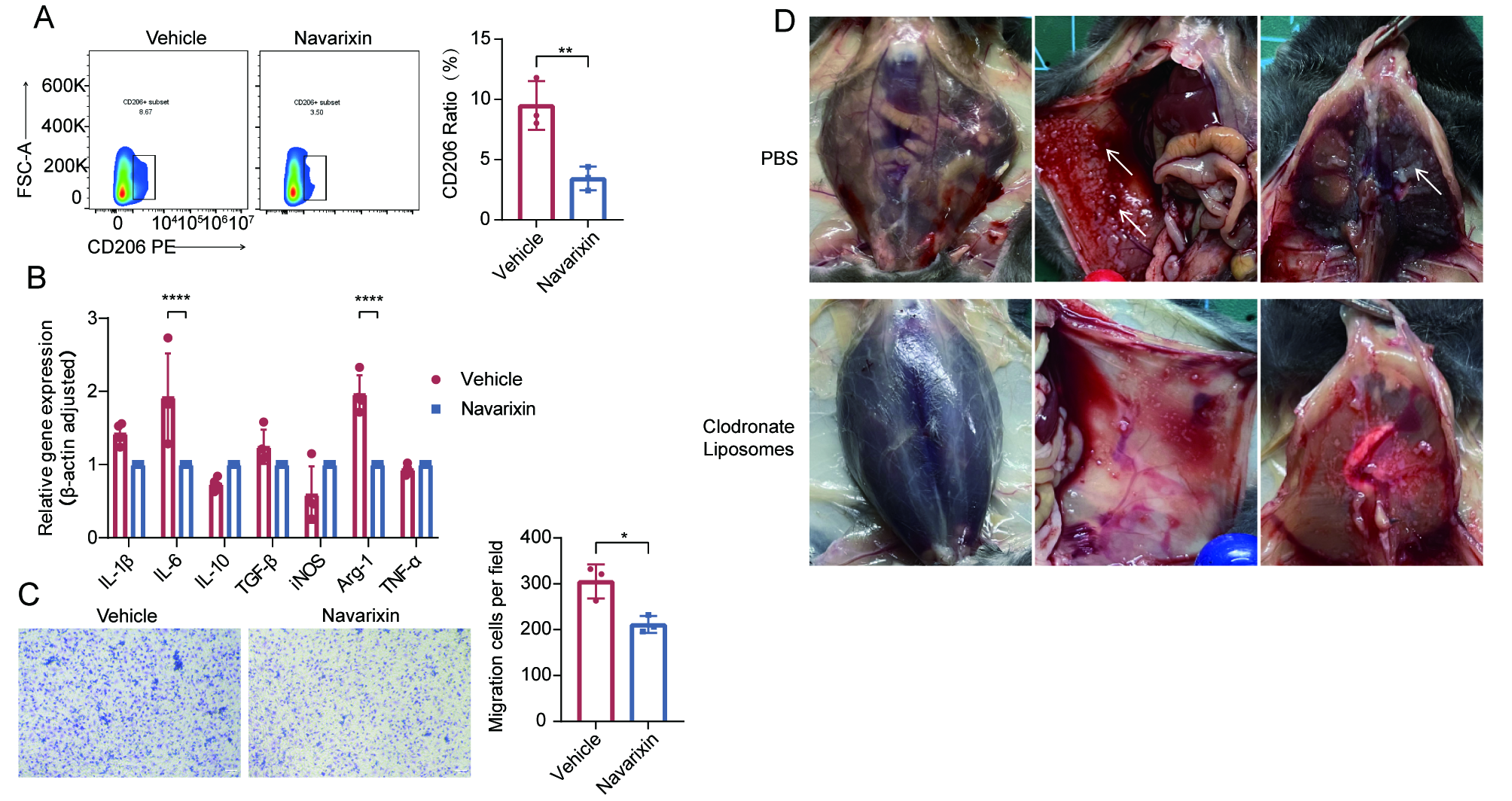

Supplement: Supplementary file 10 — Supplementary Figure 6 [file 41419_2025_8312_MOESM10_ESM.tif]
